# Bridging local–global transmembrane protein contexts with contrastive pretraining for alignment-free pathogenicity prediction

**DOI:** 10.1093/bib/bbag352

**Published:** 2026-07-03

**Authors:** Yihang Bao, Zhe Liu, Fangyi Zhao, Wenhao Li, Hui Jin, Guan Ning Lin

**Affiliations:** Shanghai Mental Health Center, Shanghai Jiao Tong University School of Medicine, School of Biomedical Engineering, Shanghai Jiao Tong University, 1954 Huashan Road, Xuhui District, Shanghai 200030, China; Engineering Research Center of Digital Medicine of the Ministry of Education, 1954 Huashan Road, Xuhui District, Shanghai 200030, China; Shanghai Gener Arfysica Intelligent Healthcare & Brain Science Research Institute, 377 Chengpu Road, Fengxian District, Shanghai 200030, China; Department of Computer Science and Engineering, 130 Meilong Road, Xuhui District, East China University of Science and Technology, Shanghai 200030, China; Shanghai Mental Health Center, Shanghai Jiao Tong University School of Medicine, School of Biomedical Engineering, Shanghai Jiao Tong University, 1954 Huashan Road, Xuhui District, Shanghai 200030, China; Shanghai Mental Health Center, Shanghai Jiao Tong University School of Medicine, School of Biomedical Engineering, Shanghai Jiao Tong University, 1954 Huashan Road, Xuhui District, Shanghai 200030, China; Shanghai Mental Health Center, Shanghai Jiao Tong University School of Medicine, School of Biomedical Engineering, Shanghai Jiao Tong University, 1954 Huashan Road, Xuhui District, Shanghai 200030, China; Shanghai Gener Arfysica Intelligent Healthcare & Brain Science Research Institute, 377 Chengpu Road, Fengxian District, Shanghai 200030, China; Shanghai Mental Health Center, Shanghai Jiao Tong University School of Medicine, School of Biomedical Engineering, Shanghai Jiao Tong University, 1954 Huashan Road, Xuhui District, Shanghai 200030, China; Engineering Research Center of Digital Medicine of the Ministry of Education, 1954 Huashan Road, Xuhui District, Shanghai 200030, China; Shanghai Gener Arfysica Intelligent Healthcare & Brain Science Research Institute, 377 Chengpu Road, Fengxian District, Shanghai 200030, China

**Keywords:** deep learning, protein language models, variant pathogenicity prediction, transmembrane proteins, contrastive-learning

## Abstract

Predicting the pathogenic consequences of protein mutations is a cornerstone of precision medicine, yet it remains a formidable challenge for transmembrane proteins (TMPs), a clinically vital class of drug targets. Existing computational methods are often hampered by their reliance on evolutionary data and fail to model TMP-specific biophysical constraints. Here, we introduce Memo-Patho, a deep learning framework for robust, alignment-free pathogenicity prediction of TMP variants. The core innovation is a within-protein, label-informed supervised contrastive pretraining strategy that learns sequence-encoded biophysical signatures distinguishing pathogenic and benign variants by directly comparing them within the same protein context. By fusing sequence-level representations from protein language models with local structural proxies derived from sequence, Memo-Patho achieves accurate predictions without multiple sequence alignments or experimental structures. Across diverse TMP benchmarks and under protein-level group splits, Memo-Patho consistently outperforms leading predictors, achieving up to 0.93 accuracy, and it transfers to an independent KCNQ1 ion-channel cohort without re-training. Its resource-efficient, alignment-free design enables routine large-scale screening when evolutionary or structural data are sparse. Conceptually, Memo-Patho addresses a key gap by directly learning discriminative, sequence-anchored signatures pertinent to TMP-specific constraints, offering a principled and generalizable foundation for research-use clinical variant triage and proteome-wide mutation-effect modeling.

## Introduction

The functional repertoire of a cell is executed by its proteins, whose roles are determined by their unique amino acid sequences and resulting three-dimensional structures [1]. Variations within these protein sequences, frequently involving the substitution of just a single amino acid residue, are common across the human population. However, even such seemingly minor alterations can profoundly disrupt protein behavior. The classic example is the Glu6Val substitution in hemoglobin’s beta chain, which leads to sickle cell anemia through altered protein solubility and aggregation [2]. Considering the vast unexplored mutational space [3], reliably predicting the functional and clinical impact of amino acid changes remains a central challenge in molecular biology and is crucial for advancing precision medicine-oriented diagnostics.

Transmembrane proteins (TMPs), comprising ~20%–30% of the human proteome [4], mediate essential transport and signaling functions within the lipid bilayer and constitute a major class of disease genes and drug targets [5, 6]. However, TMPs present significant experimental hurdles: their inherent hydrophobicity complicates expression, purification, structural determination, and functional analysis compared to soluble proteins [7]. This relative scarcity of experimental data underscores the urgent need for robust computational methods specifically tailored to predict variant effects in this vital, yet experimentally challenging, protein class.

Computational prediction of variant effects has progressed from early conservation-based approaches to modern deep learning frameworks. Representative early tools such as SIFT [8] and PROVEAN [9] relied primarily on evolutionary conservation, whereas more recent predictors have incorporated richer sequence-based, structure-based, or learning-based representations. Examples include general variant effect predictors such as MutPred2 [10], protein language model (PLM)-integrated models such as E-SNPs&GO [11], structure-informed predictors such as AlphaMissense [12, 13], and TMP-oriented methods including MutTMPredictor [14], mCSM-membrane [15], and Pred-MutHTP [16]. Despite these advances, accurate pathogenicity prediction for transmembrane proteins remains challenging because many existing methods are not designed to explicitly capture the membrane-specific structural and biophysical constraints that shape TMP variant effects.

However, many contemporary high-performing predictors still rely on generating multiple sequence alignments (MSAs) for evolutionary context or require protein structural data. These dependencies create major bottlenecks in computational cost, scalability, and data availability, especially for challenging protein classes such as TMPs. By contrast, PLMs, trained on massive protein sequence databases, can learn rich sequence representations that capture structural and functional information directly from single sequences without explicit alignments. PLM-based methods have already shown strong utility across diverse bioinformatics tasks. For example, SignalP 6.0, by leveraging the PLM ProtTrans [17], was the first to achieve comprehensive prediction across five types of signal peptides [18]. This ability to derive informative features directly from sequence makes PLMs a promising foundation for developing more scalable, generalizable, and alignment-free pathogenicity prediction tools.

Although PLMs have opened new opportunities for sequence-based variant interpretation, their application to pathogenicity prediction remains less mature, particularly for transmembrane proteins. E-SNPs&GO [11] and TransEFVP [19] incorporate PLM embeddings for mutation effect prediction and demonstrate the value of sequence-derived contextual representations. However, these methods generally treat PLM features as generic sequence descriptors and do not explicitly model mutations as local perturbations embedded within the broader structural and functional context of TMPs. Moreover, PLM-based representation learning remains limited in predictors specifically designed for TMP variant interpretation, and membrane-specific biophysical constraints are still insufficiently explored. In particular, existing PLM-integrated predictors generally do not model TMP variants as perturbations constrained by membrane topology, membrane-proximal transition zones, and the coupling between local mutation effects and broader transmembrane protein context.

Here, we address the unmet need that most TMP variants lack reliable structures and that existing predictors insufficiently capture membrane-specific biophysical constraints. We hypothesize that PLMs enable accurate, MSA-free pathogenicity prediction. This is because PLMs capture both long-range contextual dependencies and residue-level physicochemical regularities, which, when combined with local structural proxies, are sufficient to determine a variant’s impact. Guided by this rationale, we design Memo-Patho around three principles: alignment-free representation, local–global decomposition, and within-protein contrastive learning. Concretely, our contributions are: (i) removal of MSA dependence, enabling efficient, scalable screening across extensive mutational landscapes; (ii) a dual-stream use of PLM embeddings as global (contextual) and local (site-specific) representations fused by a multi-head Transformer to judge variants in their native microenvironment; (iii) contrastive pretraining that pairs benign and pathogenic variants on the same sequence to learn decision-critical, TMP-aware biophysical signatures; and (iv) integration of predicted structural descriptors to reflect how mutations perturb local topology and environment. Extensive internal and external evaluations show state-of-the-art accuracy and robust generalization to unseen proteins, establishing Memo-Patho as both a practical screening tool and a conceptually novel framework for TMP variant interpretation.

## Results

### The Memo-Patho framework

**Figure 1:**
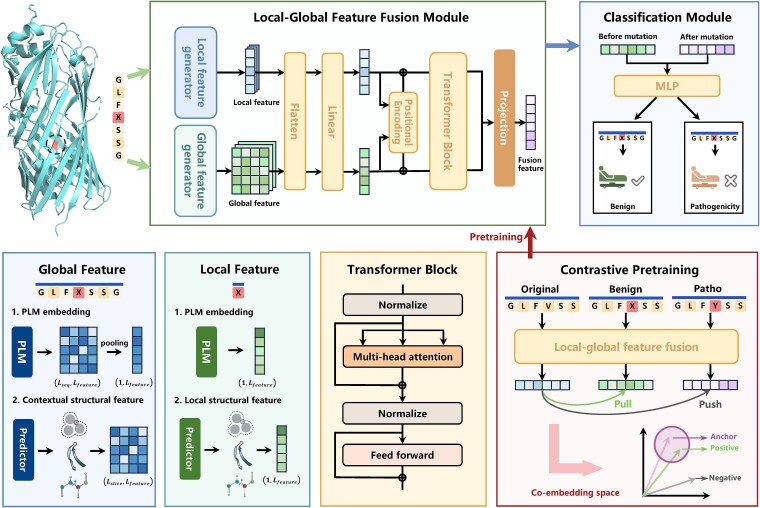
Schematic overview of the Memo-Patho architecture.

Memo-Patho is a deep learning architecture designed for the alignment-free prediction of pathogenicity associated with mutations in TMPs. Figure 1 shows the model structure. Its core principle involves integrating the localized impact of a mutation with its broader implications within the global protein structure and function. The architecture utilizes a dual-stream feature extraction process: (i) the Global Feature stream leverages PLMs to derive sequence embeddings, which are subsequently pooled to summarize global sequence information. This is combined with a contextual structural feature derived from the mutation-centered residue neighborhood to form a comprehensive global feature vector representing broader protein context; (ii) concurrently, the Local Feature stream focuses on the mutation site, extracting sequence and structural features specifically at this locus using PLMs and structure predictors. A dedicated Local–Global Feature Fusion Module then synergistically processes these features. Both local and global feature vectors undergo flattening and linear transformation. Positional encoding is added to retain sequential or spatial information before they are fed into a Transformer block. This block, via its multi-head self-attention mechanism [20], dynamically weighs feature elements and models complex interdependencies between the local mutation site and the global protein context. The output from the Transformer is passed through a final projection layer, yielding a unified representation.

A distinguishing characteristic of Memo-Patho is its contrastive pretraining strategy, employed prior to fine-tuning. This supervised contrastive pretraining phase enhances the discriminative capability of the feature fusion module. Using triplets comprising an anchor (original sequence), a positive (known benign variant), and a negative (known pathogenic variant), the model is trained via a contrastive loss function. This objective encourages the fused representations of anchor and positive samples to converge in the embedding space, while simultaneously pushing the representations of anchor and negative samples apart. This pretraining step effectively guides the model to discern subtle, pathogenicity-related feature differences within the same protein sequence.

Finally, a multilayer perceptron classifier processes this unified representation to output a final binary classification, predicting whether the mutation is benign or pathogenic. By integrating local and global contexts through a sophisticated fusion mechanism enhanced by contrastive pretraining, Memo-Patho offers a robust approach for accurate, alignment-free pathogenicity prediction in TMPs.

### Efficient mutation pathogenicity prediction using Memo-Patho and comparison with external tools

**Figure 2:**
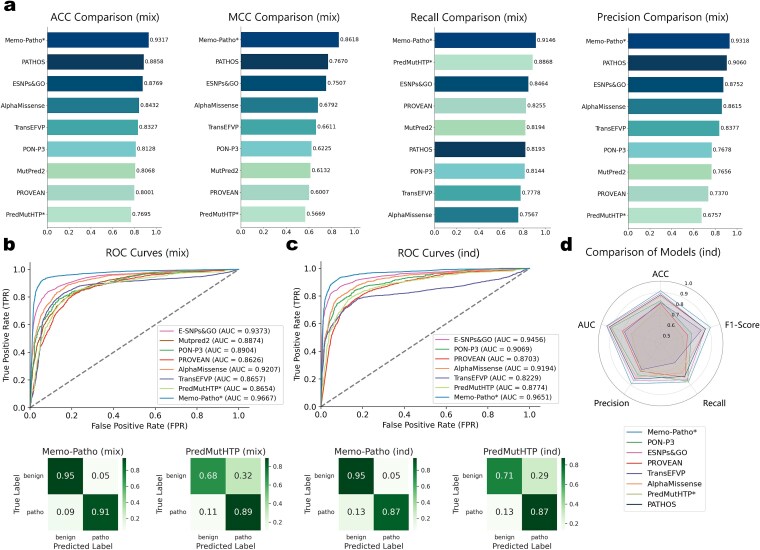
Memo-Patho performance evaluation. (a, b) Performance comparison of Memo-Patho with comparable tools on the Mix dataset. (c, d) Performance comparison of Memo-Patho with comparable tools on the Ind dataset. Note that “*” indicates the model is specific to TMPs.

We designed the evaluation to directly test our central hypothesis that an alignment-free, local-plus-global PLM representation with contrastive pretraining can improve TMP pathogenicity prediction while generalizing to proteins not seen during training. We developed and evaluated Memo-Patho using two distinct dataset partitioning strategies, termed Mix and Ind datasets, with comprehensive details on data splitting and the two-stage model training (contrastive pretraining followed by binary supervised classification) available in the Supplementary Note. Hyperparameters were selected exclusively on training folds via 10-fold cross-validation and then fixed prior to assessment on held-out independent test sets to prevent information leakage (cross-validation results are presented in Supplementary Figs 5–8 and 18). We benchmarked Memo-Patho against a suite of contemporary pathogenicity prediction tools, including TransEFVP [19], MutPred2 [10], PROVEAN [21], AlphaMissense [12], PredMutHTP (specific to TMPs) [16], ESNPs&GO [11], PON-P3 [22], and PATHOS [23]. Tool-specific settings and evaluation protocols followed the procedures detailed in the Supplementary Note to ensure metric comparability across baselines. Notably, for AlphaMissense and PON-P3, which can output an “ambiguous” classification for predictions near a probability of .5, we resolved these into benign or pathogenic categories using a .5 probability threshold for consistent comparison, unless explicitly stated otherwise. On the Mix dataset’s independent test set, Memo-Patho achieved a superior accuracy of 0.9317 and a Matthews correlation coefficient (MCC) of 0.8618 (Fig. 2a and b, Supplementary Table 1). Similarly, when evaluated on the Ind dataset’s independent test set, which assesses generalization to proteins unseen during training, Memo-Patho demonstrated robust performance with an accuracy of 0.9149 and an MCC of 0.8209, again outperforming the other tools (Fig. 2c and d, Supplementary Table 2). Notably, different baseline methods ranked differently across metrics because these measures capture distinct aspects of classifier behavior. For example, some tools favored higher recall at the cost of increased false positives, which consequently reduced their precision and MCC. These results indicate high discriminative performance when training and testing share proteins (Mix) and, critically, strong generalization to novel proteins (Ind). This advantage was not uniformly distributed across TMP topology, but was enriched in membrane-sensitive regions such as the TM core and peri-transmembrane boundary, where Memo-Patho showed the largest gains over TransEFVP on the Ind test set (Supplementary Figs 13 and 14). Together, the findings support our premise that sequence-encoded biophysical signatures combined with local structural proxies suffice for accurate TMP pathogenicity prediction without alignments. To further assess whether this advantage is biologically meaningful beyond aggregate classification metrics, we additionally evaluated protein-level pathogenic variant prioritization. As shown in Supplementary Fig. 12, Memo-Patho consistently achieved higher protein-level macro Recall@K than TransEFVP on both the Mix and Ind datasets, indicating improved prioritization of truly pathogenic variants within the same TMP rather than only better binary classification. We next asked whether this gain was enriched in TMP-relevant structural contexts. Region-stratified analysis showed that the performance improvement over TransEFVP was concentrated in topology-sensitive and membrane-proximal regions, particularly the TM core, peri-TM non-core residues, and related TM-boundary schemes (Supplementary Fig. 13). These patterns suggest that Memo-Patho is better aligned with TMP-specific structural constraints than generic PLM embedding-based predictors.

Because we cannot exclude the possibility that some external tools may have been trained on variants overlapping with our test sets, these baseline results should be interpreted with appropriate caution. Under this caveat, Memo-Patho still showed a consistent advantage, suggesting that the observed performance gain is unlikely to be overstated. Beyond its predictive accuracy, Memo-Patho also offers substantial computational efficiency due to its alignment-free architecture. This efficiency primarily arises from avoiding the expensive preprocessing steps required by alignment- or structure-dependent pipelines, allowing Memo-Patho to learn directly from PLM-derived representations and sequence-derived structural descriptors. To quantify this advantage more rigorously, we performed a dedicated two-stage runtime benchmark that separately evaluated feature generation and downstream prediction under controlled hardware settings. On 10% subsets derived from the Mix and Ind mutation spaces, Memo-Patho completed feature generation in 12.5 min and 11.2 min, respectively, compared with 324.5 min and 176.0 min for HHblits [24] and more than 1440 min for AlphaMissense feature preparation. In the subsequent prediction stage, Memo-Patho required less than 1 min on both subsets, whereas AlphaMissense required 767.8 min and 500.1 min, respectively. These results demonstrate that Memo-Patho is highly practical for high-throughput variant interpretation and large-scale mutational screening. Detailed benchmarking procedures, hardware settings, and runtime definitions are provided in the Supplementary Note.

### Enhancing prediction with effective representation learned by contrastive pretraining

To assess the effectiveness of our contrastive pretraining strategy, we investigated its capacity to learn more discriminative feature representations, beyond its impact on final classification performance. We hypothesized that this pretraining phase would refine the embedding space, making benign and pathogenic variants more separable (specific methodologies for this analysis are detailed in Methods). The theoretical motivation is that contrastive objectives learned within the same protein sequence control for background sequence context, enlarge the decision margin between pathogenic and benign variants, and encourage features that capture mutation effects conditional on the native protein environment rather than protein identity. Accordingly, we examined how the geometry of the learned embeddings changed before and after contrastive pretraining, independent of the final classification head.

**Figure 3:**
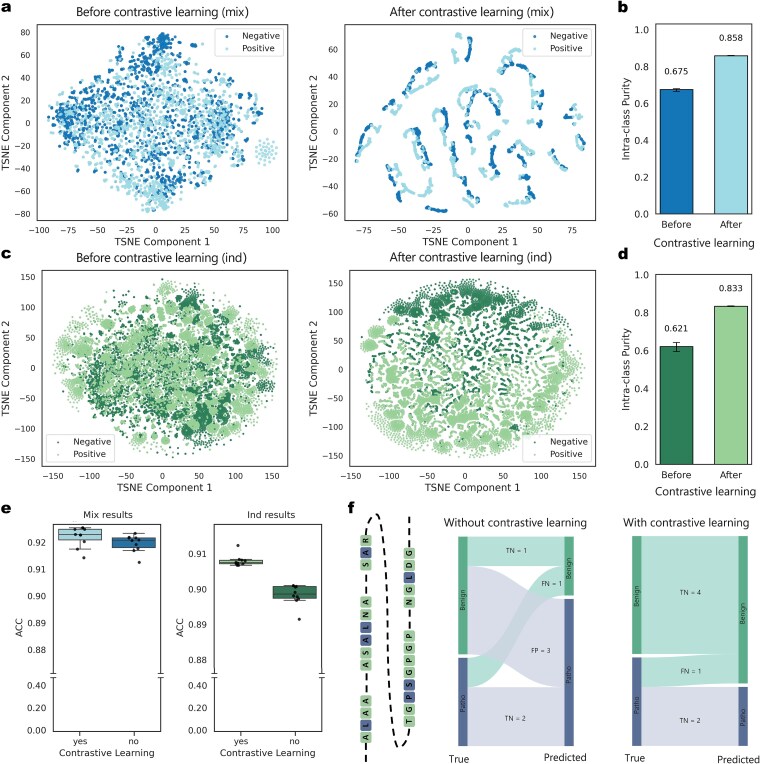
Effectiveness analysis of contrastive learning. (a) t-SNE visualization of input and output embeddings on the Mix dataset. (b) Comparison of intra-class purity on the Mix dataset before and after contrastive learning. (c) t-SNE visualization of input and output embeddings on the Ind dataset. (d) Comparison of intra-class purity on the Ind dataset before and after contrastive learning. (e) Comparison of Memo-Patho with and without contrastive learning pretraining. (f) After contrastive learning, Memo-Patho can effectively handle the complex scenario of varying mutational effects within the same sequence.

To qualitatively assess this, we employed t-distributed stochastic neighbor embedding (t-SNE) [25] for dimensionality reduction and visualization of the feature embeddings. Figure 3a and c show that contrastive pretraining yields a more structured embedding space, with benign and pathogenic variants forming more distinct clusters in both Mix and Ind datasets. Complementing this visual inspection, we conducted a quantitative analysis by evaluating intra-cluster purity. We applied a clustering algorithm to the feature embeddings both before and after contrastive pretraining and then assessed the purity of the resulting clusters with respect to the ground-truth pathogenicity labels. As depicted in Fig. 3b (for the Mix dataset) and Fig. 3d (for the Ind dataset), there is a marked improvement in intra-cluster purity in the representations obtained after contrastive pretraining compared to the initial, pre-contrastive features. This significant increase in purity provides quantitative evidence that the contrastive pretraining stage successfully enhances the discriminative power of the learned features, aligning them more closely with the inherent biological distinctions between pathogenic and benign mutations. The concordant purity gains across both partitioning schemes indicate that contrastive pretraining reduces label mixing in feature space and aligns representation structure with biological ground truth.

Following the analysis of feature representation quality, we directly investigated how contrastive pretraining contributed to the final performance of Memo-Patho. As shown in Fig. 3e, the inclusion of the contrastive pretraining stage resulted in improved performance metrics during 10-fold cross-validation on both the Mix and Ind datasets. Notably, the performance uplift appeared more significant on the Ind dataset. The gain was particularly pronounced on the Ind dataset, which evaluates generalization to unseen proteins, supporting that within-protein contrastive learning encourages robust, sequence-level features and improves out-of-distribution performance.

To further illustrate this practical benefit, Fig. 3f provides a case study from the Ind dataset’s independent test set, focusing on the protein Q6ZMI3. This protein has three mutations annotated as pathogenic and four as benign in our test data. When Memo-Patho was trained without the contrastive pretraining stage, it incorrectly predicted four of these seven variants. However, upon incorporating contrastive pretraining, the model’s accuracy for this specific protein improved to only one of the seven variants being misclassified. This example concretely illustrates how contrastive pretraining stabilizes predictions for novel targets and reduces protein-level errors in prospective screening scenarios.

### Analysis of local–global feature effectiveness revealing the importance of multi-scale features

**Figure 4:**
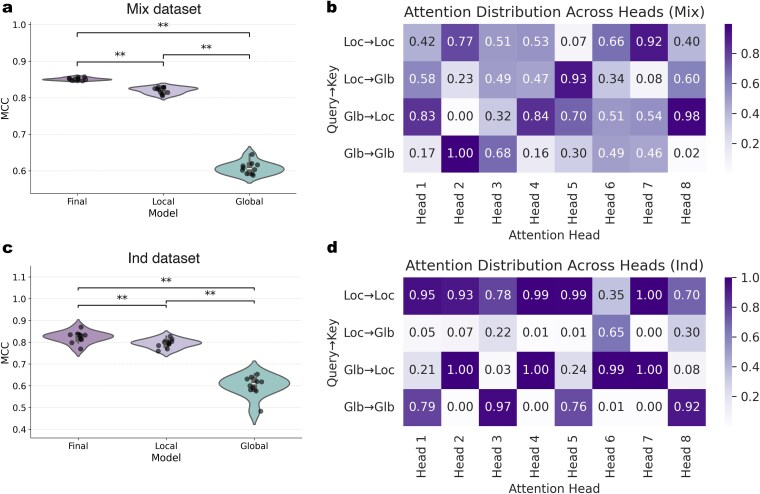
Effectiveness analysis of local–global features. (a) Comparison of Memo-Patho with its variants using only local features and only global features on the Mix dataset. (b) Attention visualization on the Mix dataset. (c) Comparison of Memo-Patho with its variants using only local features and only global features on the Ind dataset. (d) Attention visualization on the Ind dataset.

We analyzed how local and global features contribute to Memo-Patho to address whether TMP mutation effects require information from both the residue-level microenvironment and the whole-protein context. We performed ablation studies by comparing the full Memo-Patho model against versions trained using only local features or only global features. Figure 4a and c illustrate the 10-fold cross-validation performance of these models on the Mix and Ind datasets, respectively. The results clearly demonstrate that both local features (derived from the immediate vicinity of the mutation) and global features (representing the broader protein context) are individually effective in predicting mutation pathogenicity. Crucially, across both partitioning schemes, the combined model consistently outperforms either single-source variant, indicating true complementarity rather than redundancy and supporting our multi-scale design, where accurate TMP variant interpretation benefits from explicit integration of site-specific signals and protein-level context. We further evaluated whether the performance gain also depended on combining the two protein language models. We also tested a smaller PLM backbone, ESM2-650M, and observed performance comparable to the ESM2-only variant on both datasets, without a significant difference (Supplementary Fig. 9). This suggests that the framework remains effective under smaller PLM configurations and may therefore be adapted for more speed-sensitive applications. Ablation experiments showed that the full model using both ESM2 and ProtT5 consistently outperformed the ESM2-only and ProtT5-only variants on both the Mix and Ind datasets, supporting that the two PLMs provide complementary information rather than redundant sequence representations (Supplementary Fig. 9).

To further understand how local and global features are integrated, we analyzed the attention mechanisms within the feature fusion stage of the mutation processing branch. We extracted the attention coefficients from eight attention heads, examining interactions categorized as local-to-local, local-to-global, global-to-local, and global-to-global. Across datasets, attention mass was enriched for local information, with local-to-local and local-to-global interactions receiving the largest weights during fusion. The results, depicted in Fig. 4b (Mix dataset) and Fig. 4d (Ind dataset), reveal that local features generally command greater attention during the fusion process. This finding aligns with our ablation experiments, which highlighted the strong predictive capacity of local features. We also observe a systematic shift between datasets: on the Ind split that enforces generalization to unseen proteins, the model increases its reliance on local evidence, whereas on Mix, it allocates relatively more weight to broad contextual cues. This pattern is theoretically consistent with our design premise that the residue-level signal remains stable under distribution shift, while global context is more beneficial when some elements of the protein background overlap with training. Together, these analyses provide mechanism-aligned evidence that multi-scale fusion is necessary for robust TMP pathogenicity prediction and not merely an additive combination of features. Consistently, an inference-time perturbation analysis showed that removing mutation-centered local branches caused the largest performance drop, particularly in the TM core and peri-transmembrane boundary, further supporting that Memo-Patho’s advantage is primarily driven by local TMP-sensitive representations (Supplementary Fig. 15).

### Enabling high-throughput interpretation of protein mutation pathogenicity using Memo-Patho

**Figure 5:**
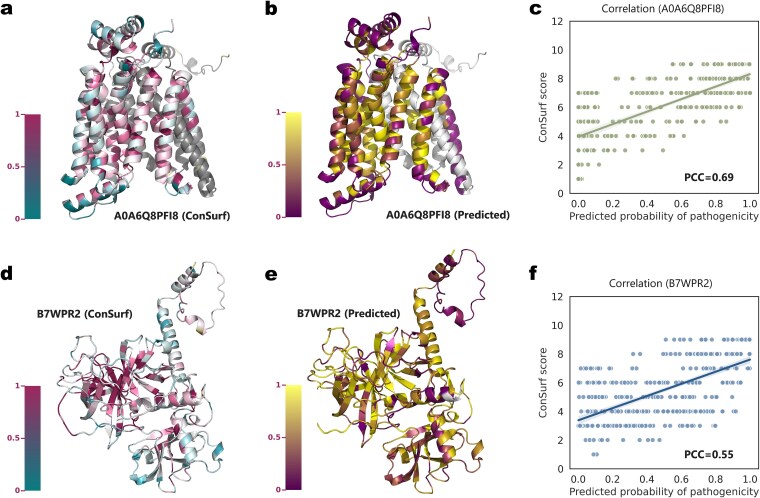
Correlation analysis with conservation. (a, b) Visualization of conservation scores and Memo-Patho prediction scores on A0A6Q8PFI8. (c) Correlation plot of conservation scores versus Memo-Patho prediction scores on A0A6Q8PFI8. (d, e) Visualization of conservation scores and Memo-Patho prediction scores on B7WPR2. (f) Correlation plot of conservation scores versus Memo-Patho prediction scores on B7WPR2.

We set out to demonstrate not only that Memo-Patho scales to proteome-level workloads, but also that its predictions recover biologically meaningful constraints in the absence of alignments or experimental structures, which is essential for practical variant triage. The alignment-free architecture of Memo-Patho underpins its capacity for rapid processing of extensive sequence datasets, positioning it as an exceptionally suitable tool for large-scale mutational pathogenicity screening. To test both throughput and biological relevance under realistic conditions, we performed exhaustive *in silico* mutational scans on two held-out human proteins with distinct lengths and functions, A0A6Q8PFI8 (359 residues) and B7WPR2 (Transmembrane Serine Protease 3, 451 residues). We then investigated the relationship between Memo-Patho’s predictions and sequence conservation. Sequence conservation, reflecting the evolutionary pressure to maintain specific amino acids at positions critical for protein structure or function, serves as an independent biological indicator of residue importance. Mutations at highly conserved sites are generally more likely to be deleterious [26]. The detailed methodology for calculating conservation scores and performing this comparison is provided in Methods. For protein A0A6Q8PFI8, the distribution of site-specific conservation scores (Fig. 5a) and Memo-Patho’s average predicted pathogenicity scores per residue (Fig. 5b) demonstrate a compelling visual correspondence. This relationship was quantified with a Pearson correlation coefficient of 0.69 (Fig. 5c), signifying a strong positive correlation. Similar strong correlations and visual concordance were observed for B7WPR2 (Fig. 5d–f). Because conservation information is not used by the model, these correlations show that Memo-Patho recovers evolutionary constraints from sequence-encoded signals alone, supporting the theoretical premise that PLM-based local and global representations can substitute for explicit alignments. Taken together, the concordance between predicted pathogenicity landscapes and conservation argues that the model prioritizes residues with functional or structural indispensability, thereby increasing confidence in variant prioritization when experimental data are sparse.

The comprehensive mutational scan for A0A6Q8PFI8 and B7WPR2 generated a dataset of 15 390 unique amino acid substitutions. Memo-Patho processed this entire dataset, delivering pathogenicity predictions for 0.31 seconds per variant, enabling routine scanning of full mutational neighborhoods around candidate sites. In practice, these proteome-scale maps can guide experimental design by flagging high-value regions for mutagenesis, inform clinical curation by prioritizing variants of uncertain significance (VUS), and support target assessment by revealing sensitive positions in membrane proteins. Together, the combined throughput and biological fidelity position Memo-Patho as a scalable engine for hypothesis generation and decision support in TMP-focused research and beyond.

### Memo-Patho unveils functional impact of critical KCNQ1 variants

**Figure 6:**
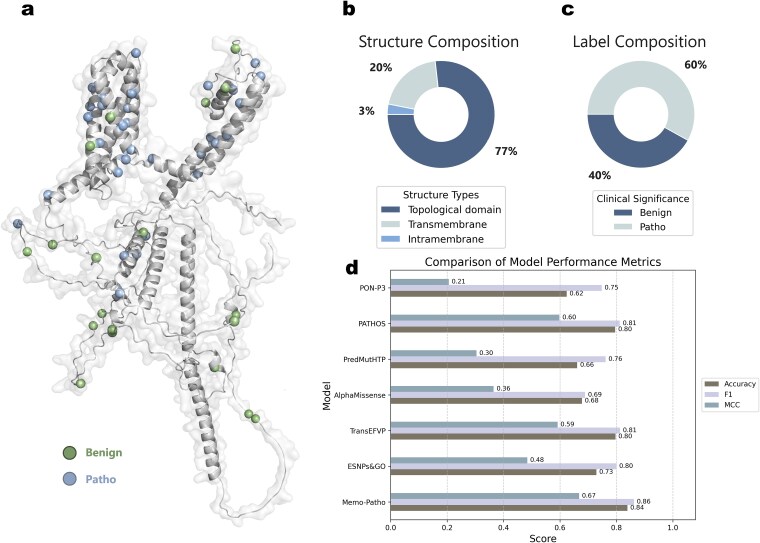
Performance comparison on the novel dataset. (a) Visualization of mutations on the novel KCNQ1 dataset. (b) Distribution of topological structure types for KCNQ1. (c) Label distribution on the novel mutation dataset. (d) Predictive performance comparison of Memo-Patho with other state-of-the-art tools.

To further validate Memo-Patho’s effectiveness and its ability to generalize to entirely independent data, we conducted testing on a novel dataset of variants in the KCNQ1 protein. This dataset was recently established by Brewer et al. (published 19 February 2025) and comprises 93 variants [27] sourced from databases such as HGMD [28], including those with known, controversial, or previously undetermined clinical significance. The KCNQ1 gene encodes a critical voltage-gated potassium channel, and its loss-of-function is the predominant cause of Type 1 Long QT Syndrome (LQTS1), one of the most prevalent inherited cardiac arrhythmogenic disorders [29]. The study by Brewer et al. involved a comprehensive suite of experimental analyses, meticulously probing the functional, trafficking, and biophysical properties of these variants to definitively ascertain their pathogenicity. From this dataset, we excluded variants that overlapped with our own training and initial test sets, resulting in a stringent novel test set of 55 KCNQ1 mutation records. The structural distribution of these 55 mutations is depicted in Fig. 6a, while their distribution across various structural regions and the composition of their assigned pathogenic/benign labels are detailed in Fig. 6b and c, respectively. For this evaluation, all model hyperparameters, probability calibration, and decision thresholds were fixed from internal validation and were not adjusted on the KCNQ1 data, ensuring a strictly out-of-sample assessment.

When evaluated on this challenging KCNQ1 dataset, Memo-Patho demonstrated superior predictive performance compared to other contemporary tools, achieving an accuracy of 0.84 and an MCC of 0.67 (Fig. 6d, Supplementary Fig. 17, and Supplementary Table 3). Performance remained consistent when variants were stratified by structural regions, supporting the conclusion that the model captures transferable signals beyond protein identity. Notably, the improvement over AlphaMissense was not uniformly distributed across KCNQ1 regions, but was more pronounced in the intracellular regulation and coupling region than in other regions (Supplementary Fig. 11). It is consistent with the TMP-focused design of Memo-Patho and suggests improved sensitivity to region-specific functional contexts. This strong performance on a rigorously characterized, clinically relevant, and novel protein dataset underscores that the model has learned transferable principles of variant pathogenicity rather than overfitting to its training set. In practical terms, this external validation demonstrates transportability to an independent, expert-curated cohort, which is a prerequisite for downstream clinical interpretation. The ability to accurately classify variants in a well-studied disease gene such as KCNQ1 suggests that Memo-Patho can serve as a powerful adjunctive tool for prioritizing VUS for further experimental investigation, potentially accelerating diagnostic workflows and improving the understanding of disease mechanisms in clinically actionable genes. More broadly, the KCNQ1 analysis illustrates how Memo-Patho can be integrated into variant curation pipelines to rank candidate substitutions, flag discordant cases for targeted assays, and generate mechanistic hypotheses for follow-up.

## Discussion

Accurately predicting protein mutation pathogenicity is pivotal for genomics and precision medicine, yet existing methods often struggle with generalization, dependence on time-consuming MSAs, and limited scalability for large-scale screening. Many clinically actionable variants, especially in TMPs, lack reliable evolutionary alignments or experimental structures, restricting current predictors. We posit that large protein language models encode long-range contextual constraints and residue-level regularities which, when combined with local structural proxies and a within-protein contrastive objective, enable alignment-free pathogenicity inference. Guided by this premise, Memo-Patho integrates sequence and predicted structural features via contrastive pretraining followed by supervised fine-tuning to achieve accurate, generalizable, and computationally efficient predictions.

Memo-Patho directly addresses key challenges in variant interpretation. Its alignment-free design removes the MSA bottleneck, while contrastive pretraining sensitizes the model to subtle mutational effects on identical protein backbones. Combined with rich representations from protein language models (ESM2, ProtT5) and predicted structural properties (NetSurfP-3.0), Memo-Patho learns robust, generalizable features well-suited for large-scale genomic screening.

Methodologically, our primary innovations are an explicitly alignment-free representation strategy, a local and global decomposition fused by attention, and a within-protein contrastive learning scheme that controls for background sequence context. To our knowledge, this combination has not been systematically applied to TMP pathogenicity prediction and provides a principled mechanism to learn biophysical signatures directly from sequence. This interpretation was further supported by stratified analyses showing that Memo-Patho achieved its largest relative gains on mutation classes that disrupt membrane-relevant biophysical constraints, including hydrophobic-to-charged substitutions, charge introduction within TM segments, and helix-breaking changes (Supplementary Fig. 16).

The development of robust predictors like Memo-Patho holds particular significance for TMPs. These proteins are integral to cellular physiology, serve as crucial drug targets, and are frequently implicated in disease, yet pathogenic annotations for TMPs are relatively scarce compared to globular proteins, potentially leading to their underrepresentation in general predictors. Consequently, such models may not fully capture unique TMP constraints. Memo-Patho’s strong performance, achieved without MSA reliance and supported by effective feature learning, offers a focused approach to address this gap and improve TMP variant interpretation.

Memo-Patho’s strategies yield state-of-the-art performance and robust generalization on our TMP-centric Mix and Ind datasets and superior performance on an externally curated KCNQ1 variant dataset. Given KCNQ1’s pivotal role in cardiac function, these results underscore that Memo-Patho learns transferable rules beyond individual protein identity, supporting TMP-related clinical applications such as triage of VUS and prioritization for targeted assays. In addition, Memo-Patho showed encouraging probability calibration on the independent protein split, with a Brier score of 0.0744 and an expected calibration error (ECE) of 0.0554, supporting its potential utility as a decision-support tool for TMP-focused variant prioritization. This advantage was further preserved across proteins with different sequence lengths, as shown by the sequence-length-stratified analysis in Supplementary Fig. 20.

Our analyses confirm the synergistic value of local and global features. Attention mechanisms generally prioritized local features, particularly for novel proteins in the Ind dataset, while selectively incorporating global TMP context. Together with the refined feature space induced by contrastive pretraining, these findings provide mechanism-aligned evidence that multi-scale fusion is essential for robust TMP pathogenicity prediction.

Memo-Patho’s biological relevance is further supported by strong positive correlations between its predictions and evolutionary conservation scores (e.g. Pearson coefficient of 0.69 for A0A6Q8PFI8, with similar findings for TMPRSS3/B7WPR2), despite conservation not being used as input. This suggests that evolutionary constraints are being recovered from sequence-encoded signals alone, reinforcing the validity of the alignment-free design.

A key advantage of Memo-Patho is its computational efficiency: its alignment-free architecture processes thousands of variants orders of magnitude faster than MSA-based approaches. Although integrating both ESM2 and ProtT5 increases the computational cost at the embedding extraction stage, this overhead was moderate relative to the performance gain, while the downstream classifier inference time remained negligible across PLM configurations (Supplementary Table 4). This makes it highly practical for comprehensive *in silico* mutational scans across the transmembrane proteome, enabling rapid prescreening, calibration on internal controls, and iterative refinement of candidate lists for experimental validation within realistic compute budgets. Notably, Memo-Patho also showed strong within-protein prioritization ability, with high protein-level Recall@K (Supplementary Fig. 10).

Despite its robust performance, Memo-Patho shares limitations common to current deep learning-based variant effect predictors. Although attention analysis provides a coarse view of feature weighting, the mechanistic basis of individual predictions remains only partially interpretable, and future work should incorporate more rigorous attribution, calibration, uncertainty quantification, and cohort-level threshold transfer analyses. Moreover, residual errors on the independent KCNQ1 cohort were concentrated in structurally challenging regions, particularly the voltage-sensing domain and pore- or coupling-adjacent sites, suggesting that these effects are not yet fully captured by the current representation (Supplementary Fig. 19). In addition, because Memo-Patho is trained on experimentally validated mutations curated from public resources such as ClinVar and UniProt, it is inevitably affected by dataset biases that are widespread in current supervised pathogenicity prediction, including unequal representation of TMP families, dominance of α-helical proteins, class-specific label imbalance, and concentration of annotations in a subset of intensively studied proteins. Our protein-level Ind setting partially alleviates this issue by testing on unseen proteins, but it cannot fully remove representation bias for underrepresented TMP subclasses. In particular, β-barrel TMPs remain sparsely represented in currently available clinically annotated variant datasets, which is a broader limitation of the field rather than a constraint unique to Memo-Patho. More comprehensive and balanced TMP variant resources, together with advances in high-throughput functional assays, will therefore be essential for further improving the robustness and generalizability of models such as Memo-Patho.

Overall, Memo-Patho represents a substantive advancement in TMP-focused pathogenicity prediction. By integrating contrastive pretraining, multi-scale attention-based fusion, and predicted structural descriptors within an alignment-free framework, the model delivers accurate, generalizable, and computationally efficient predictions. Its strengths for TMPs and its external validation on KCNQ1 indicate transportability to clinically relevant scenarios. As it is prospectively evaluated and integrated with expanding functional datasets, Memo-Patho is well positioned to support genomic research, clinical variant interpretation, and precision medicine.

## Materials and methods

### Data curation and dataset construction

Human transmembrane protein sequences were obtained from the UniProt Knowledgebase (UniProtKB, accessed 20 January 2025), a central hub for curated protein sequence and functional information [30]. Variant annotations were sourced from ClinVar, a public archive documenting relationships between human genetic variations and observed health statuses [31]. ClinVar annotations were standardized into a binary pathogenicity setting. Specifically, variants labeled as Benign or Likely benign were grouped as benign, whereas variants labeled as Pathogenic or Likely pathogenic were grouped as pathogenic. After mapping ClinVar missense annotations to the corresponding UniProt protein sequences, the initial collection comprised 13 226 proteins associated with 38 822 pathogenic and 48 347 benign variants. We then applied a multistep quality-control procedure to ensure sequence-level consistency and variant validity. First, we removed entries for which the reported wild-type residue in ClinVar did not match the residue at the corresponding position in the UniProt reference sequence. This filtering step yielded 81 884 curated mutation annotations. We subsequently performed sequence-length filtering and per-variant mutation-consistency checks, retaining only valid single-amino-acid substitutions for downstream analysis. The final curated dataset comprised 52 975 missense variants in human TMPs and served as the basis for all subsequent model development and evaluation.

### Variant-centered sequence and structural feature construction

To capture comprehensive sequence information, we utilized embeddings derived from two state-of-the-art PLMs: ESM2 and ProtT5. ESM2 is a deep transformer-based model developed by Meta AI, pretrained on vast quantities of protein sequences to learn underlying biological properties [32]. ProtT5 is an encoder-decoder transformer model from the Rost Lab, also trained on large protein datasets and adept at capturing sequence-level patterns relevant to structure and function [17]. Leveraging both models is advantageous, as prior studies suggest they can offer complementary insights into protein characteristics [19, 33]. Specific details of the embedding generation are available in the Supplementary Note. For features derived from ESM2, which produces embeddings with dimensions (sequence length × 2560), the local feature was defined as the specific embedding vector corresponding to the mutated residue position (1 × 2560). The global feature was obtained by applying mean pooling across the entire sequence’s embedding vectors, resulting in a fixed-size representation (1 × 2560). An analogous procedure was applied to the ProtT5 embeddings (output dimensions: sequence length × 1024). The local feature was extracted directly from the mutation site (1 × 1024), and the global feature was generated via mean pooling over the full sequence embedding (1 × 1024). The formula is as follows:


(1) \begin{equation*} {f}_{local, ESM2}={E}_m \end{equation*}

(2) \begin{equation*} {f}_{global, ESM2}=\frac{1}{L}\sum_{i=1}^L{E}_i \end{equation*}

(3) \begin{equation*} {f}_{local, ProtT5}={P}_m \end{equation*}

(4) \begin{equation*} {f}_{global, ProtT5}=\frac{1}{L}\sum_{i=1}^L{P}_i \end{equation*}

where $S$ is the protein sequence, $L$ is its length, $m$ is the position index of the mutated residue $\left(1\le m\le L\right)$, $ESM2(S)$ is the $L\times 2560$ embedding matrix output by the ESM2 model for sequence $S$, $ProtT5(S)$ is the $L\times 1024$ embedding matrix output by the ProtT5 model for sequence $S$, ${E}_i\in{R}^{1\times 2560}$ is the row vector within $ESM2(S)$ corresponding to the $i$-th residue, and ${P}_i\in{R}^{1\times 1024}$ is the row vector within $ProtT5(S)$ corresponding to the $i$-th residue.

Furthermore, we incorporated predicted structural features into our analysis, including relative solvent accessibility (RSA), absolute solvent accessibility (ASA), secondary structure (SS), backbone dihedral angles, and intrinsic disorder. These features were included as numerous studies have indicated direct or indirect associations between local structural characteristics and variant pathogenicity [34, 35]. To obtain these predictions, we utilized NetSurfP-3.0 [36], a prediction tool that leverages PLM embeddings, thereby avoiding computationally expensive multiple sequence alignments, and has been specifically fine-tuned on structural annotation tasks. NetSurfP-3.0 outputs a consolidated 16-dimensional feature vector per residue summarizing these predicted structural properties. For each variant, the local structural feature was defined as this 16-dimensional vector corresponding precisely to the mutated residue position. To represent the structural context, a contextual structure feature was constructed by considering a window of residues spanning 4 positions upstream and 4 positions downstream of the mutation site (9 residues in total). The 16-dimensional feature vectors for these 9 residues were then aggregated to form a 9 × 16 matrix representing the local structural environment. In cases where the mutation occurred near the sequence termini and the full 9-residue window could not be formed, the missing positions in the matrix were padded with a value of −1. The formula is as follows:


(5) \begin{equation*} {f}_{local, struct}={v}_m \end{equation*}

(6) \begin{equation*} {v}_{m+j}^{\prime }=\left\{\begin{array}{@{}l}{v}_{m+j},\kern0.5em if\ 1\le m+j\le L\\{}{p}_{pad},\kern0.5em if\ m+j<1\ or\ m+j>L\end{array}\right. \end{equation*}

(7) \begin{equation*} {f}_{global, struct}={\left[{\left({v}_{m-4}^{\prime}\right)}^T,{\left({v}_{m-3}^{\prime}\right)}^T,\dots, {\left({v}_{m+4}^{\prime}\right)}^T\right]}^T \end{equation*}

where $NSP(S)$ is the $L\times 16$ output matrix from NetSurfP-3.0 for sequence S, ${v}_i\in{R}^{1\times 16}$ is the $i$-th row vector of $NSP(S)$ representing the 16-dimensional structural feature vector for the $i$-th residue, and ${p}_{pad}\in{R}^{1\times 16}$ is a padding vector consisting entirely of −1 values.

### Dataset construction and partitioning

To evaluate Memo-Patho under both mutation-level and protein-level generalization settings, we constructed two complementary dataset partitions, termed the Mix and Ind settings. These two partitions were designed to assess different levels of difficulty in TMP variant pathogenicity prediction. The Mix setting evaluates whether the model can correctly predict unseen mutations when other variants from the same protein may still appear in the training data, whereas the Ind setting provides a stricter test of generalization by requiring prediction on proteins that are entirely unseen during training.

In the Mix setting, the dataset was partitioned at the level of individual mutation records. Specifically, 10% of all curated mutation entries were randomly sampled to form the independent test set, and the remaining 90% were used for model development, including training and validation. Under this design, a protein sequence appearing in the test set could also be present in the training/validation data, but only with different mutation entries. This setting, therefore, measures the model’s ability to generalize to unseen variants within partially familiar protein backgrounds. The resulting Mix dataset contained 47 677 training/validation entries and 5298 independent test entries.

In the Ind setting, the dataset was partitioned at the protein-sequence level. We randomly assigned 10% of the unique protein sequences, together with all of their associated variants, to the independent test set, while the remaining proteins and variants were used for training and validation. This ensured that no protein sequence in the Ind test set was encountered during model development, thereby providing a more stringent evaluation of the model’s ability to generalize to entirely novel TMPs. The resulting Ind dataset comprised 48 135 training/validation entries and 4840 independent test entries. Detailed data distributions for both partitioning schemes are provided in Supplementary Figs 1–4.

### Contrastive pretraining

We adopt a label-informed contrastive pretraining over TMP variants using curated pathogenic/benign labels. Given an anchor-positive–negative (A, P, N) construction, labels determine positives (same class) and negatives (different class). APN relations were constructed in a within-protein, label-informed manner. Contrastive relations were not pre-enumerated as a fixed triplet list, but were formed dynamically within each training batch using semi-hard mining.

#### Backbone and projection

Let $x$ denote the variant-centered input (sequence context and optional structural descriptors). The backbone encoder ${f}_{\theta }$ outputs $h={f}_{\theta }(x)$. A two-layer projection head ${g}_{\phi }$ maps to a contrastive space $z=\mathrm{normalize}\left({g}_{\phi }(h)\right)\in{\mathbb{R}}^d$, where normalize $\left(\cdotp \right)$ is ${\ell}_2$ normalization.

#### Batch-wise supervised contrastive objective

For a mini-batch $B$, define for each anchor $i$ the positive set $P(i)$ and all comparison candidates $A(i)$. The supervised contrastive loss is


(8) \begin{equation*} {\mathcal{L}}_{\mathrm{supcon}}=\sum_{i\in B}\kern0.1em \left[-\frac{1}{\left|P(i)\right|}\sum_{p\in P(i)}\kern0.20em \log \frac{\exp \left(\frac{z_i^{\top }{z}_p}{\tau}\right)}{\sum_{a\in A(i)}\kern0.20em \exp \left(\frac{z_i^{\top }{z}_a}{\tau}\right)}\right], \end{equation*}

where $\tau >0$ is the temperature. This formulation efficiently aggregates many (A, P, N) relationships within a batch.

#### Triplet-margin view (equivalent many-to-one form)

Using one positive $p(i)\in P(i)$ and one negative $n(i)\in N(i)=\left\{n\in B:{y}_n\ne{y}_i\right\}$, the triplet-margin objective is


(9) \begin{equation*} {\mathcal{L}}_{\mathrm{triplet}}=\sum_{i\in B}\kern0.1em {\left[{\left\Vert{z}_i-{z}_{p(i)}\right\Vert}_2^2-{\left\Vert{z}_i-{z}_{n(i)}\right\Vert}_2^2+m\right]}_{+}, \end{equation*}

with margin $m>0$ and ${\left[u\right]}_{+}=\max \left(u,0\right)$. In practice, we use the batch-wise supervised contrastive loss above. The triplet view is shown for clarity because our sampling is described in (A, P, N) terms. We select $p(i)$ and $n(i)$ by semi-hard mining within the batch to stabilize training.

### Analysis of representation separability before and after contrastive pretraining

To assess whether contrastive pretraining improves the separability of benign and pathogenic variants in the learned representation space, we analyzed feature distributions before and after the contrastive pretraining stage using both qualitative visualization and quantitative clustering-based evaluation. Specifically, we compared two representation levels: (i) the pre-contrastive input representation, defined as the concatenated feature vectors provided to the model before fusion, and (ii) the post-contrastive fused representation, defined as the output embedding of the fusion module after contrastive pretraining. For qualitative visualization, we applied t-SNE to both representation levels in order to project the high-dimensional feature space into two dimensions for visual inspection of class structure. The t-SNE analysis was performed using the implementation provided in the Python scikit-learn library with default parameter settings. For quantitative evaluation, we assessed intra-cluster purity with respect to the ground-truth benign/pathogenic labels. Specifically, we applied K-means clustering to the representations before and after contrastive pretraining, and then calculated the purity of the resulting clusters. To reduce dependence on any single clustering configuration, we systematically varied the number of clusters from 20 to 70 and examined whether the purity improvement after contrastive pretraining remained consistent across this range. This analysis allowed us to quantify whether contrastive pretraining led to a more label-consistent organization of the representation space.

### Correlation analysis between Memo-Patho site-level scores and evolutionary conservation

To investigate the effectiveness of Memo-Patho for large-scale mutational space screening and to assess the biological relevance of its predictions, we analyzed the correlation between its site-specific average prediction scores and evolutionary conservation scores. For this analysis, we selected two proteins, A0A6Q8PFI8 and B7WPR2/TMPRSS3, and computationally enumerated all possible single amino acid substitutions for each position within these sequences, generating a total of 15 390 unique mutation entries. Site-specific conservation scores were determined using the ConSurf web server (available at https://consurf.tau.ac.il/) [37]. The ConSurf methodology involves an initial search for homologous sequences, followed by the construction of a multiple sequence alignment using MAFFT [38]. Subsequently, an empirical Bayesian algorithm is employed to calculate evolutionary conservation scores based on the phylogenetic relationships between the sequences, classifying each site into one of nine discrete grades, where a score of 1 indicates the most variable (least conserved) positions and a score of 9 signifies the most conserved positions. After obtaining pathogenicity predictions from Memo-Patho for all 15 390 generated mutations, we calculated a single representative prediction score for each residue position by averaging Memo-Patho’s output scores for all possible mutations occurring at that specific site. Visualizations presented in Fig. 5, such as mapping these scores onto protein structures, were rendered using PyMOL [39]. The Pearson correlation coefficient, quantifying the linear relationship between the site-specific ConSurf conservation scores and Memo-Patho’s average site-specific prediction scores, was computed using the pearsonr function from the scipy.stats module in Python.

### External validation on the independent KCNQ1 dataset

For evaluation on the independent KCNQ1 dataset reported by Brewer et al. [27], we used the same testing strategy and computational pipeline as those applied to the internal Mix and Ind datasets. In particular, feature generation, model inference, and metric calculation were conducted under the same protocol, so that performance on the KCNQ1 cohort could be compared directly with the internal evaluations without introducing dataset-specific changes in preprocessing or analysis. Annotation information for KCNQ1, including sequence-based features and regional definitions used in the regional analysis (Fig. 6b), was obtained from UniProt. For structural visualization of the spatial distribution of the 55 evaluated variants (Fig. 6a), we used the full-length KCNQ1 structure predicted by AlphaFold2 [13] as a uniform structural template. This structure was used for visualization purposes only and not as an additional input to Memo-Patho. It was selected because it provides a complete full-length model, enabling consistent mapping of variant positions across the entire protein.

**Key Points**

- Introduces Memo-Patho, a dedicated deep learning framework for transmembrane proteins that fuses protein language model embeddings with multi-scale structural and physicochemical descriptors to predict missense variant pathogenicity without MSAs or experimental structures, directly addressing this data-scarce yet clinically critical protein class.
- Employs a label-informed, within-protein supervised contrastive strategy that directly contrasts pathogenic and benign variants on the same sequence scaffold, sensitizing the model to subtle, context-dependent biophysical perturbations and improving generalization by controlling for background sequence context.
- Constructs a rigorously curated, large-scale TMP variant resource and demonstrates that Memo-Patho consistently outperforms state-of-the-art predictors in accuracy, calibration, and robustness across diverse benchmark scenarios, achieving up to ~0.93 accuracy with high MCC under stringent protein-level splits.
- Validates strong generalization on independent, clinically relevant datasets, including KCNQ1 ion-channel mutations, where predictions align with evolutionary and functional evidence, highlighting its reliability for unseen proteins and real-world decision support.
- Delivers an alignment-independent, computationally efficient framework enabling scalable proteome-wide variant triage and more informed clinical and pharmacological interpretation of transmembrane protein mutations.

## List of Abbreviations

 ASA Absolute Solvent Accessibility.

RSA Relative Solvent Accessibility.

TMP / TMPs Transmembrane Protein(s).

Ind(dataset) Dataset Partitioned Independently at the Protein Level.

KCNQ1 Potassium Voltage-Gated Channel Subfamily Q Member 1.

MCC Matthews Correlation Coefficient.

Mix(dataset) Dataset Partitioned in a Mixed Manner at the Mutation-Entry Level.

MSA / MSAs Multiple Sequence Alignment(s).

PPI Protein–Protein Interaction.

VUS Variant(s) of Uncertain Significance.

PLM Protein Language Model.

## Supplementary Material

Memo-Patho_Supplementary_final_bbag352

## Data Availability

Sequence information was obtained from UniProt (https://www.uniprot.org/), and variant annotations were sourced from ClinVar (https://www.ncbi.nlm.nih.gov/clinvar/). Sequence conservation results were derived from ConSurf, with the outputs downloaded from https://consurf.tau.ac.il/. AlphaMissense prediction results were retrieved from https://alphamissense.hegelab.org via its API. KCNQ1 mutation annotation data were directly extracted from the supplementary data of the article available at https://www.pnas.org/doi/full/10.1073/pnas.2412971122. The datasets and features preprocessed in this study are available at https://github.com/RoarBoil/Memo-Patho.

## Code availability

The data and source code of Memo-Patho are available at https://github.com/RoarBoil/Memo-Patho.
