# Supplementary material for: Bridging local–global transmembrane protein contexts with contrastive pretraining for alignment-free pathogenicity prediction
Source: Brief Bioinform. 2026 Jul 3;27(4):bbag352. doi: 10.1093/bib/bbag352 (PMC13331354; doi:10.1093/bib/bbag352)
Supplement: Memo-Patho_Supplementary_final_bbag352 [file memo-patho_supplementary_final_bbag352.docx]

# Supplemental Online Content

# Memo-Patho: Bridging Local-Global Transmembrane Protein Contexts with Contrastive Pretraining for Alignment-Free Pathogenicity Prediction

Yihang Bao^a,b,c,#^, Zhe Liu ^d,#^ , Fangyi Zhao ^a,#^, Wenhao Li^a^, Hui Jin^a,c^, and Guan Ning Lin^a,b,c,^*

^a^ Shanghai Mental Health Center, Shanghai Jiao Tong University School of Medicine, School of Biomedical Engineering, Shanghai Jiao Tong University, Shanghai, China

^b^ Engineering Research Center of Digital Medicine of the Ministry of Education, Shanghai, China

^c^ Shanghai Gener Arfysica Intelligent Healthcare & Brain Science Research Institute, Shanghai, China

^d^ Department of Computer Science and Engineering, East China University of Science and Technology, Shanghai, China

*Author to whom correspondence should be addressed. (Guan Ning Lin: [nickgnlin@sjtu.edu.cn](mailto:nickgnlin@sjtu.edu.cn))

^#^ These authors contributed equally to this work

**Supplementary Figures**

1. **Supplementary Figure 1.** Label distribution across datasets.
2. **Supplementary Figure 2.** Mutation position distribution by clinical significance across datasets.
3. **Supplementary Figure 3.** Sequence length distribution across datasets.
4. **Supplementary Figure 4.** Sequence length distribution by clinical significance across datasets.
5. **Supplementary Figure 5.** Confusion matrix of Memo-Patho's 10-fold cross-validation results on the Mix dataset.
6. **Supplementary Figure 6.** Radar chart of Memo-Patho's 10-fold cross-validation results on the Mix dataset.
7. **Supplementary Figure 7.** Confusion matrix of Memo-Patho's 10-fold cross-validation results on the Ind dataset.
8. **Supplementary Figure 8.** Radar Chart of Memo-Patho's 10-fold cross-validation results on the Ind dataset.
9. **Supplementary Figure 9.** Performance of Memo-Patho under different PLM backbone sizes.
10. **Supplementary Figure 10.** Within-protein variant prioritization performance measured by protein-level macro Recall@K.
11. **Supplementary Figure 11.** Region-specific performance gain of Memo-Patho over AlphaMissense on KCNQ1.
12. **Supplementary Figure 12.** Protein-level Recall@K of Memo-Patho and TransEFVP.
13. **Supplementary Figure 13.** Region-specific gain of Memo-Patho over TransEFVP.
14. **Supplementary Figure 14.** Representative TMP families with Memo-Patho gain over TransEFVP.
15. **Supplementary Figure 15.** Feature perturbation analysis of Memo-Patho on the Ind dataset.
16. **Supplementary Figure 16.** Memo-Patho advantage across TMP biophysical constraint strata.
17. **Supplementary Figure 17.** KCNQ1 variants corrected by Memo-Patho but missed by TransEFVP.
18. **Supplementary Figure 18.** Hyperparameter sensitivity analysis of Memo-Patho across different learning rates and batch sizes, evaluated by MCC
19. **Supplementary Figure 19.** Distribution of residual misclassified variants of Memo-Patho across structural regions in the independent KCNQ1 dataset.
20. **Supplementary Figure 20.** Length-stratified performance comparison of Memo-Patho and TransEFVP.

**Supplementary Notes**

1. PLMs embedding generation.
2. Calculation methods for evaluation metrics.
3. Evaluation workflow for comparable tools.
4. Memo-Patho training methods and settings.
5. Runtime benchmarking and implementation details.

**Supplementary Tables**

1. ***Supplementary Table 1.*** *Performance evaluation between baseline models on Mix dataset*
2. ***Supplementary Table 2.*** *Performance evaluation between baseline models on Ind dataset*
3. ***Supplementary Table 3.*** *Performance evaluation between baseline models on novel dataset*
4. ***Supplementary Table 4.*** *Computational efficiency of different PLM configurations*
5. ***Supplementary Table 5.*** *Runtime benchmark of Memo-Patho and representative baseline pipelines under controlled hardware settings*

Supplementary Figure


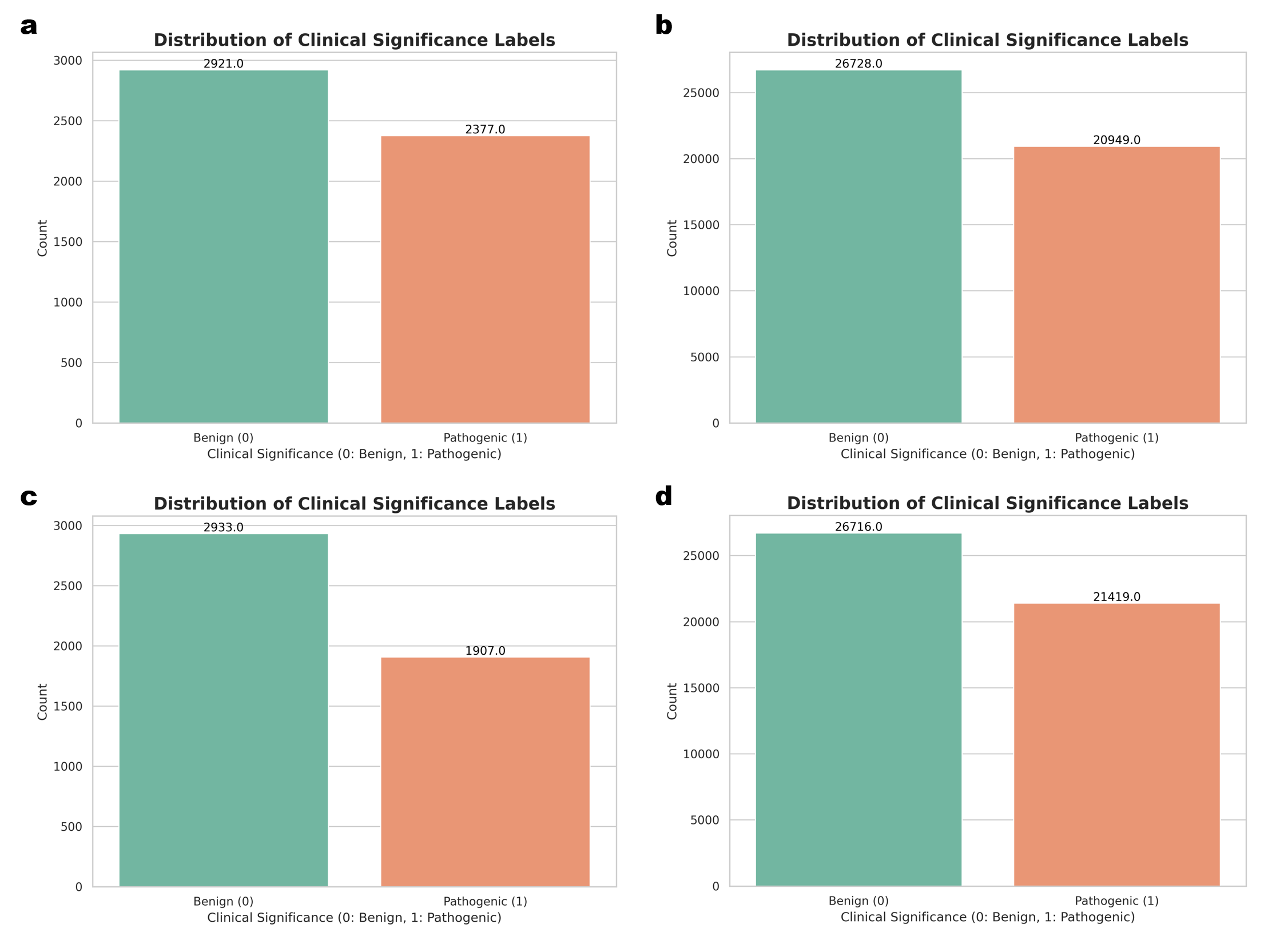


**Supplementary Figure 1.** Label distribution across datasets. **(a)** Mix test set. **(b)** Mix train set. **(c)** Ind test set. **(d)** Ind train set.


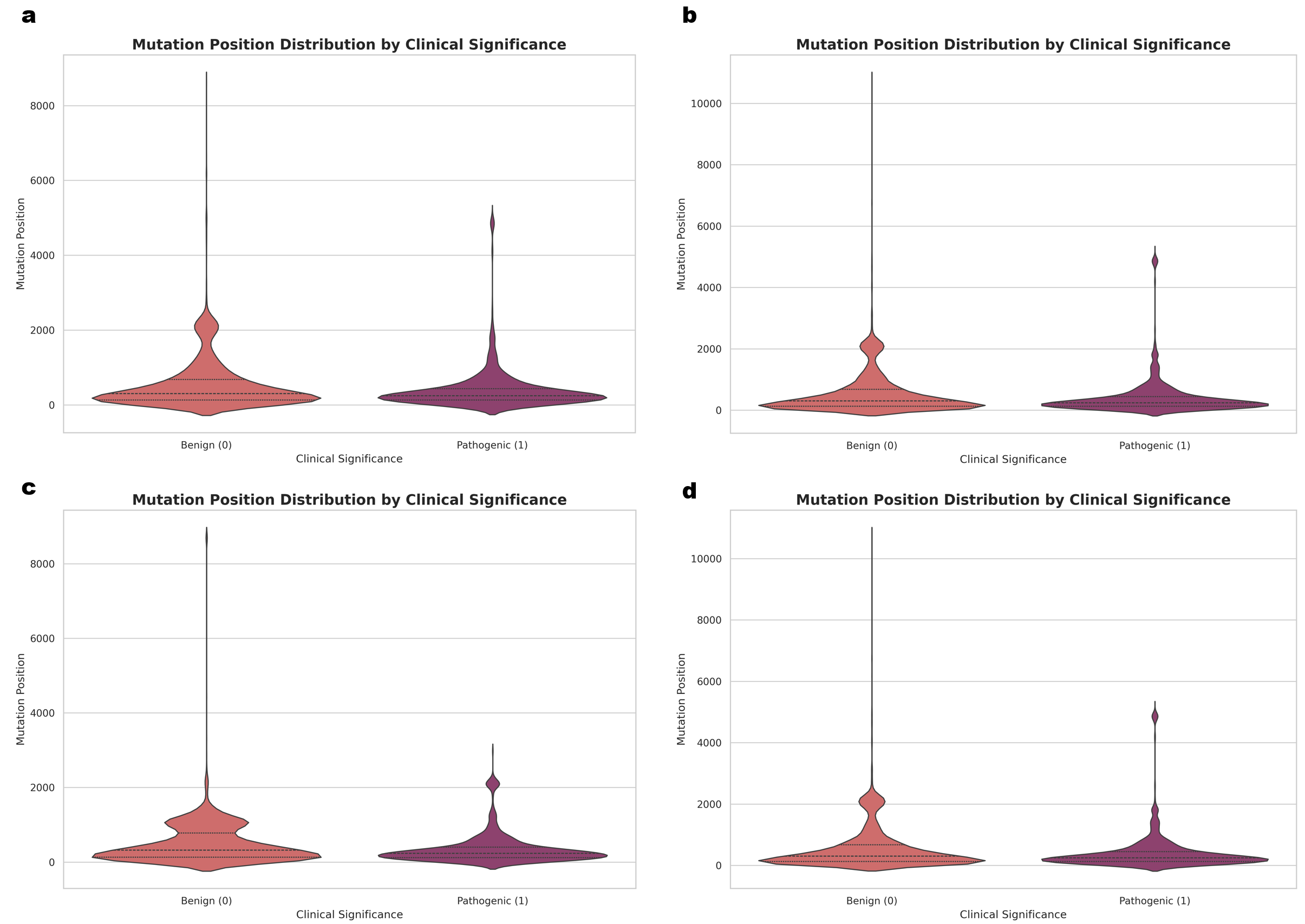


**Supplementary Figure 2.** Mutation position distribution by clinical significance across datasets. **(a)** Mix test set. **(b)** Mix train set. **(c)** Ind test set. **(d)** Ind train set.


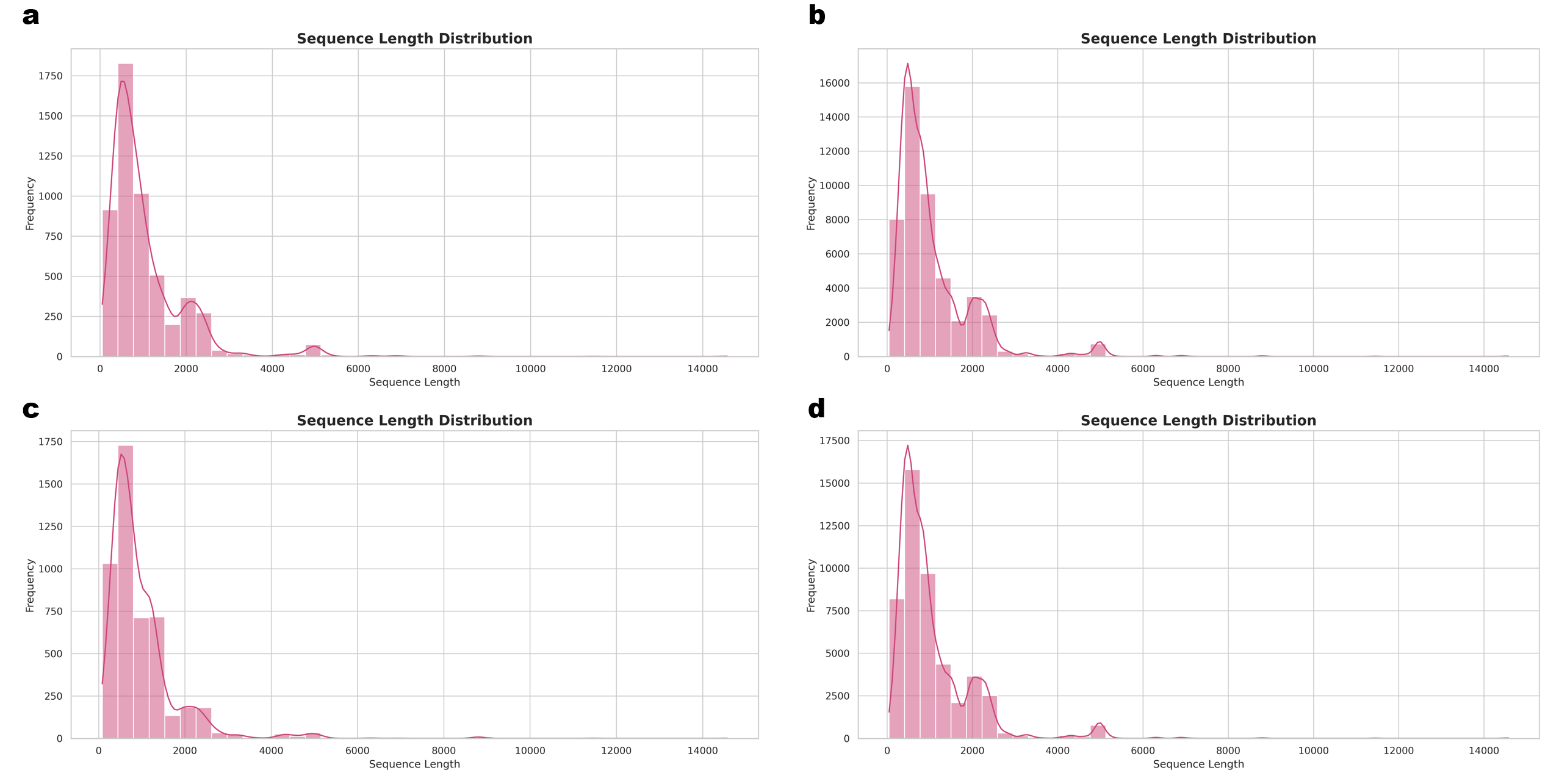


**Supplementary Figure 3.** Sequence length distribution across datasets. **(a)** Mix test set. **(b)** Mix train set. **(c)** Ind test set. **(d)** Ind train set.


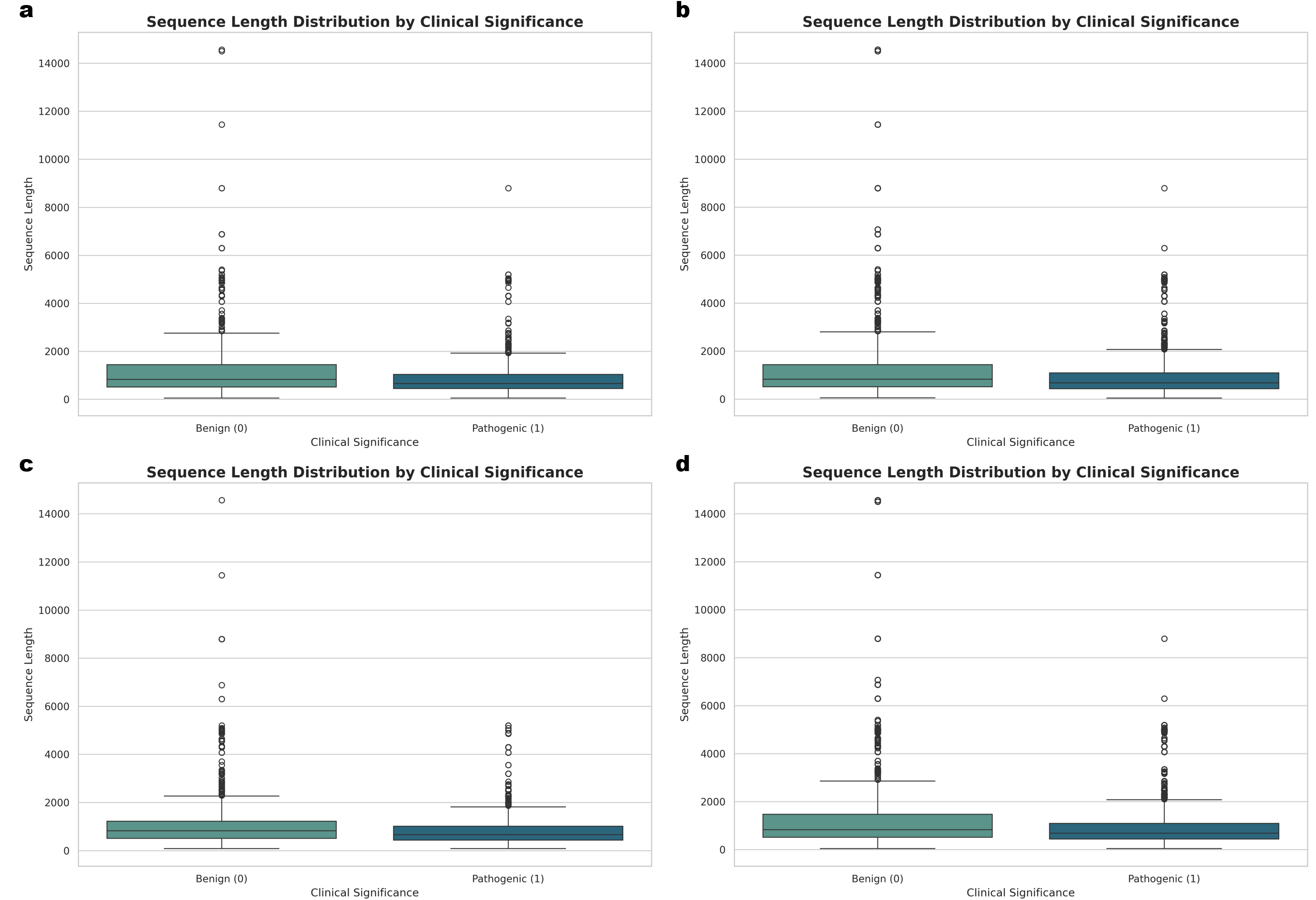


**Supplementary Figure 4.** Sequence length distribution by clinical significance across datasets. **(a)** Mix test set. **(b)** Mix train set. **(c)** Ind test set. **(d)** Ind train set.


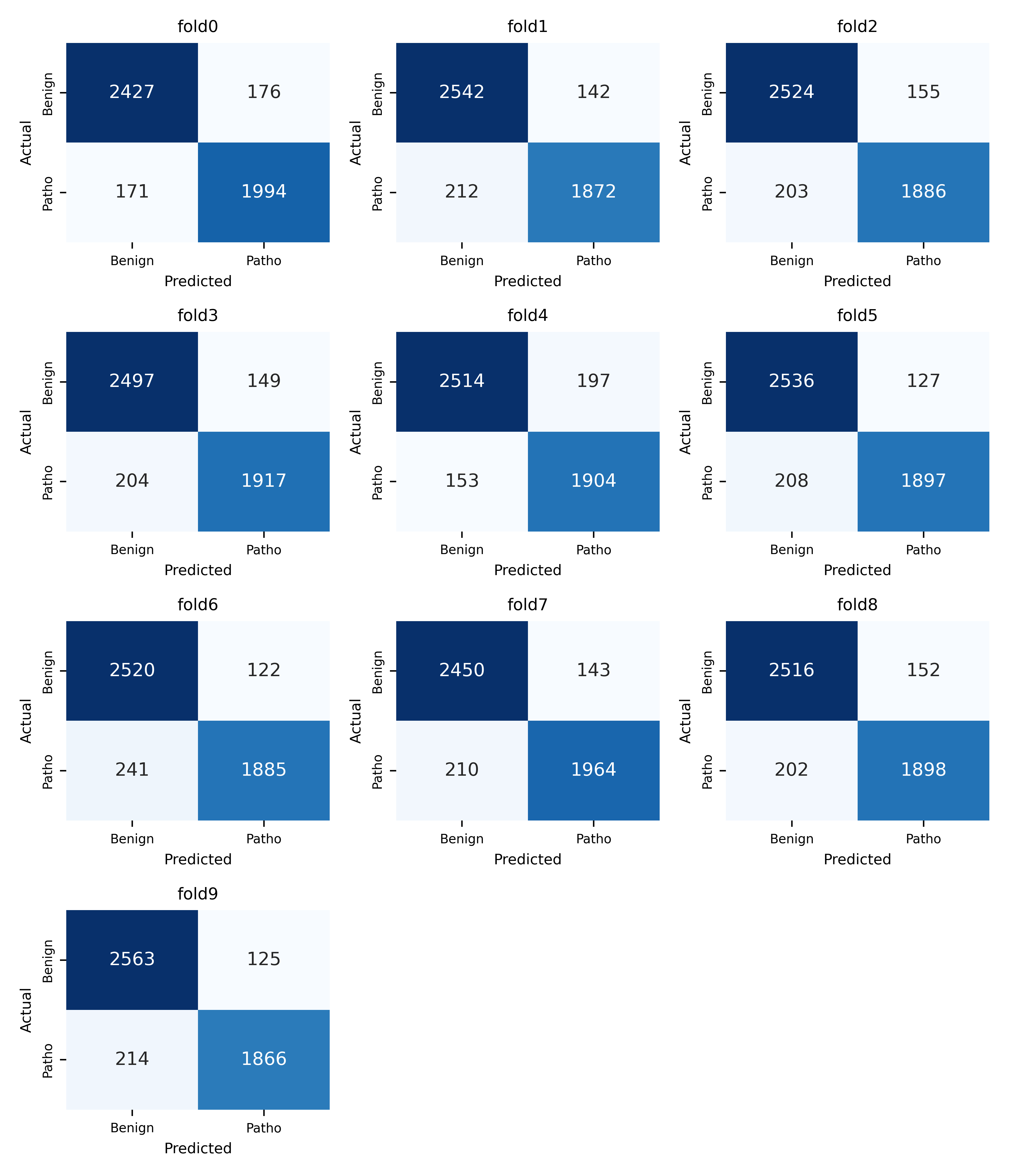


**Supplementary Figure 5.** Confusion matrix of Memo-Patho's 10-fold cross-validation results on the Mix dataset.


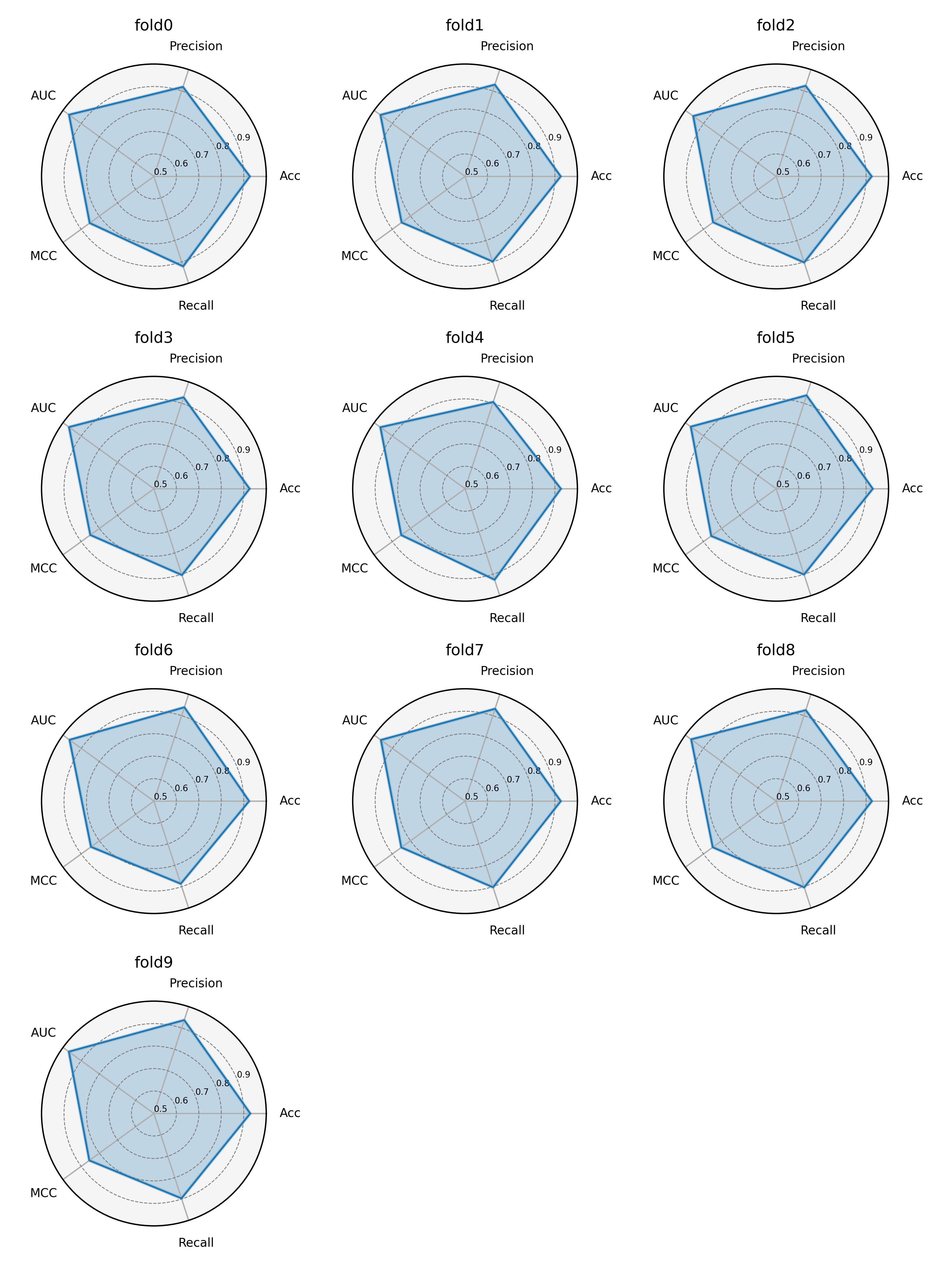


**Supplementary Figure 6.** Radar chart of Memo-Patho's 10-fold cross-validation results on the Mix dataset.


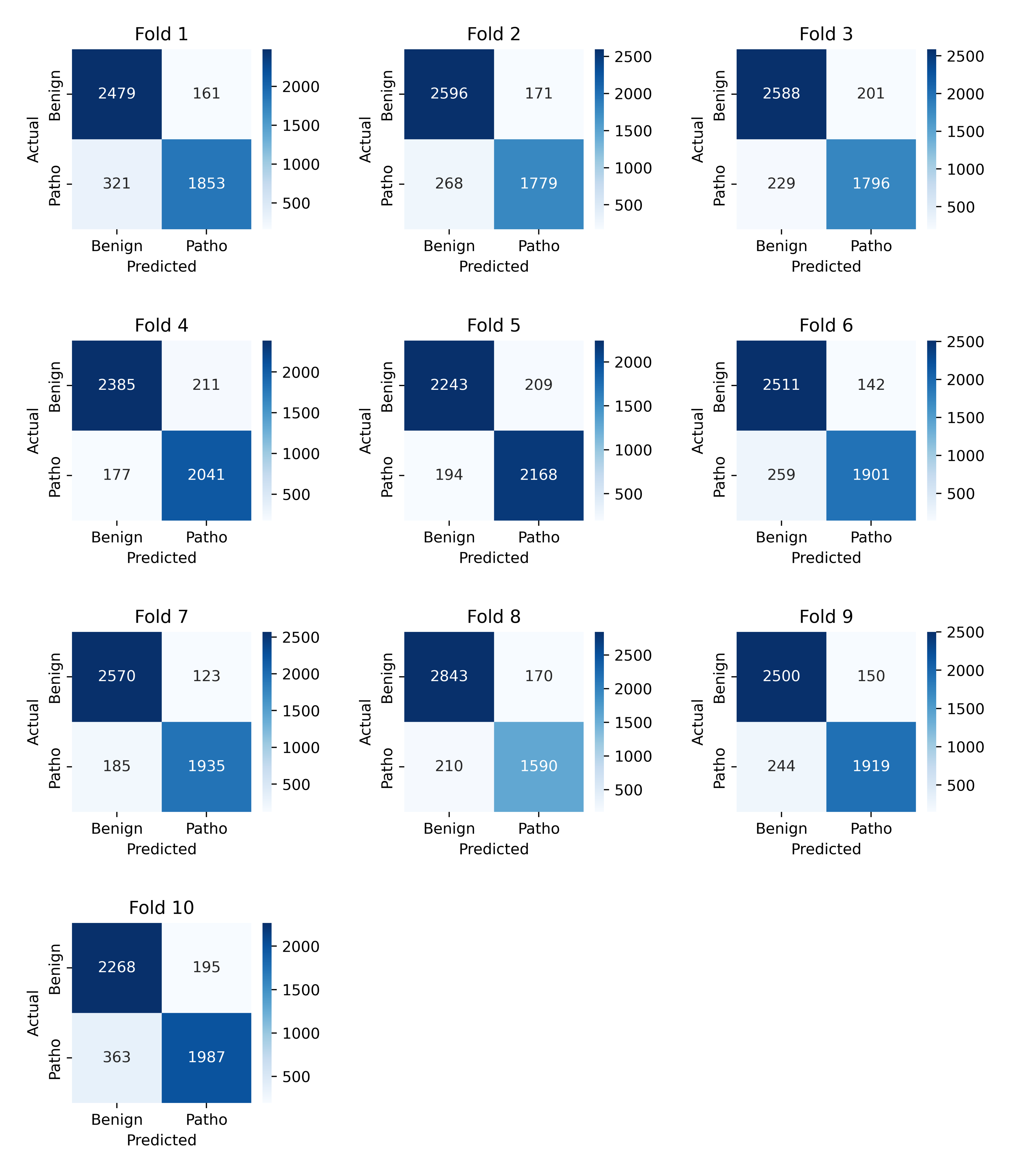


**Supplementary Figure 7.** Confusion matrix of Memo-Patho's 10-fold cross-validation results on the Ind dataset.


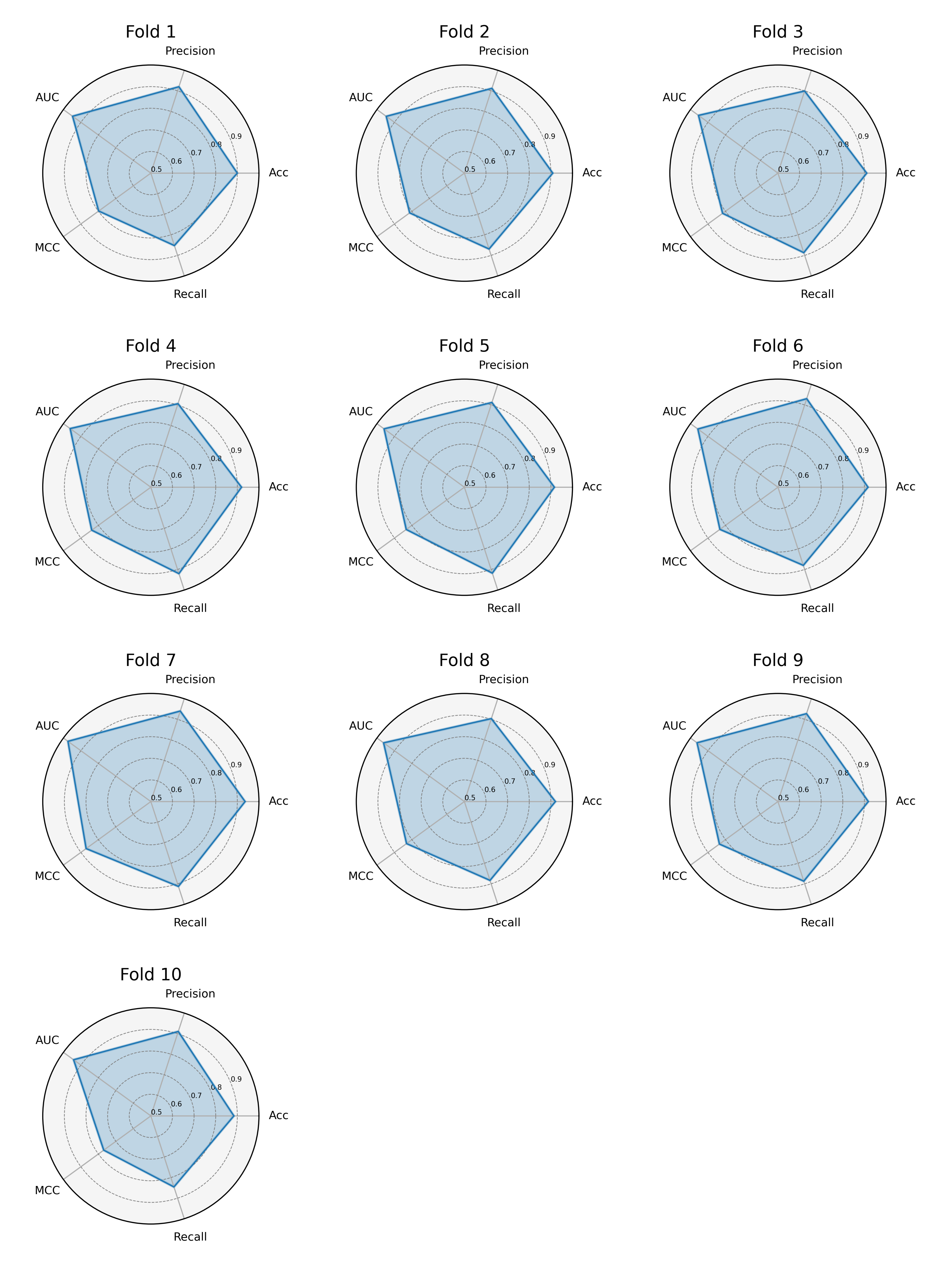


**Supplementary Figure 8.** Radar Chart of Memo-Patho's 10-fold cross-validation results on the Ind dataset.


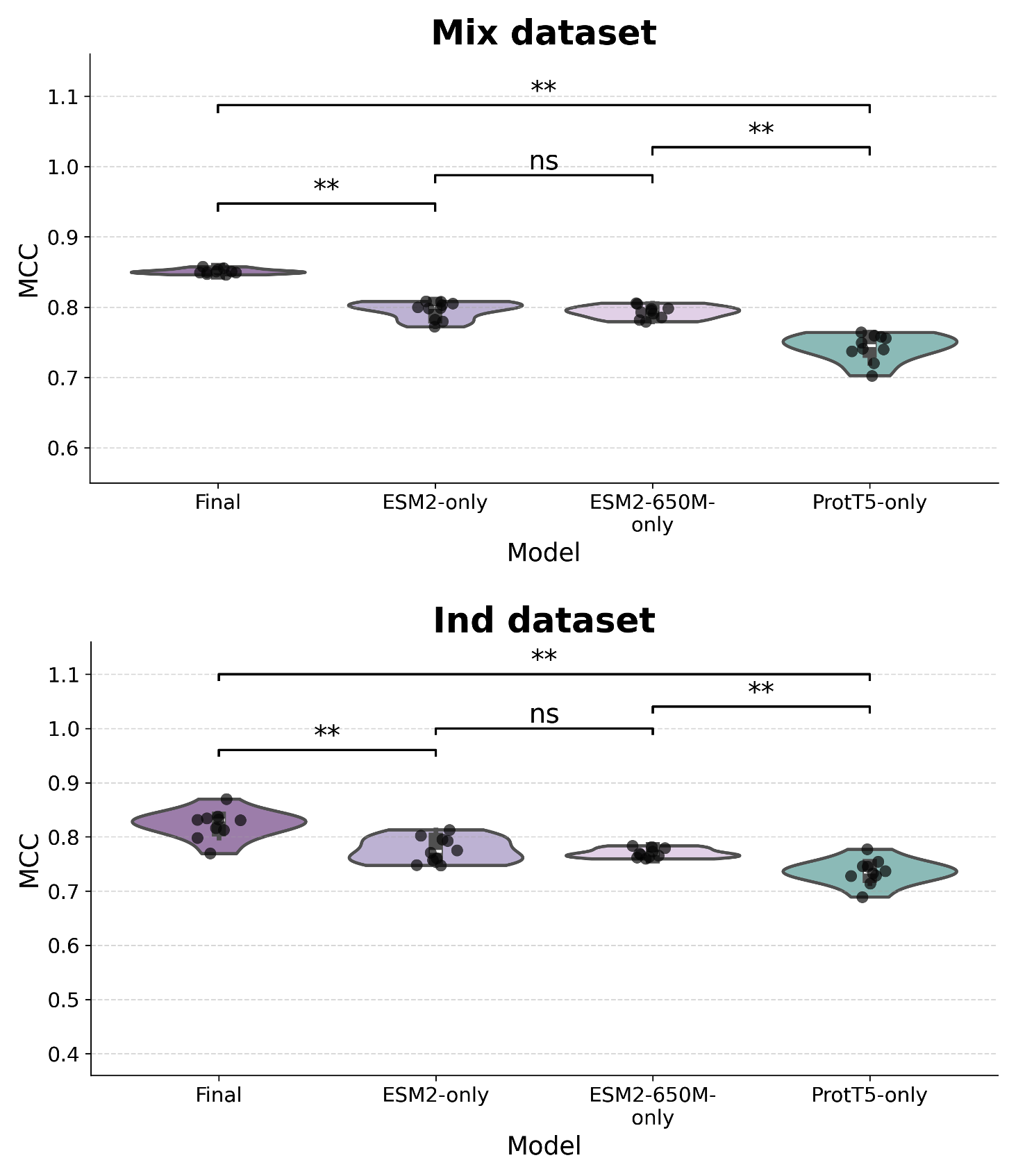


**Supplementary Figure 9.** Performance of Memo-Patho under different PLM backbone sizes. **
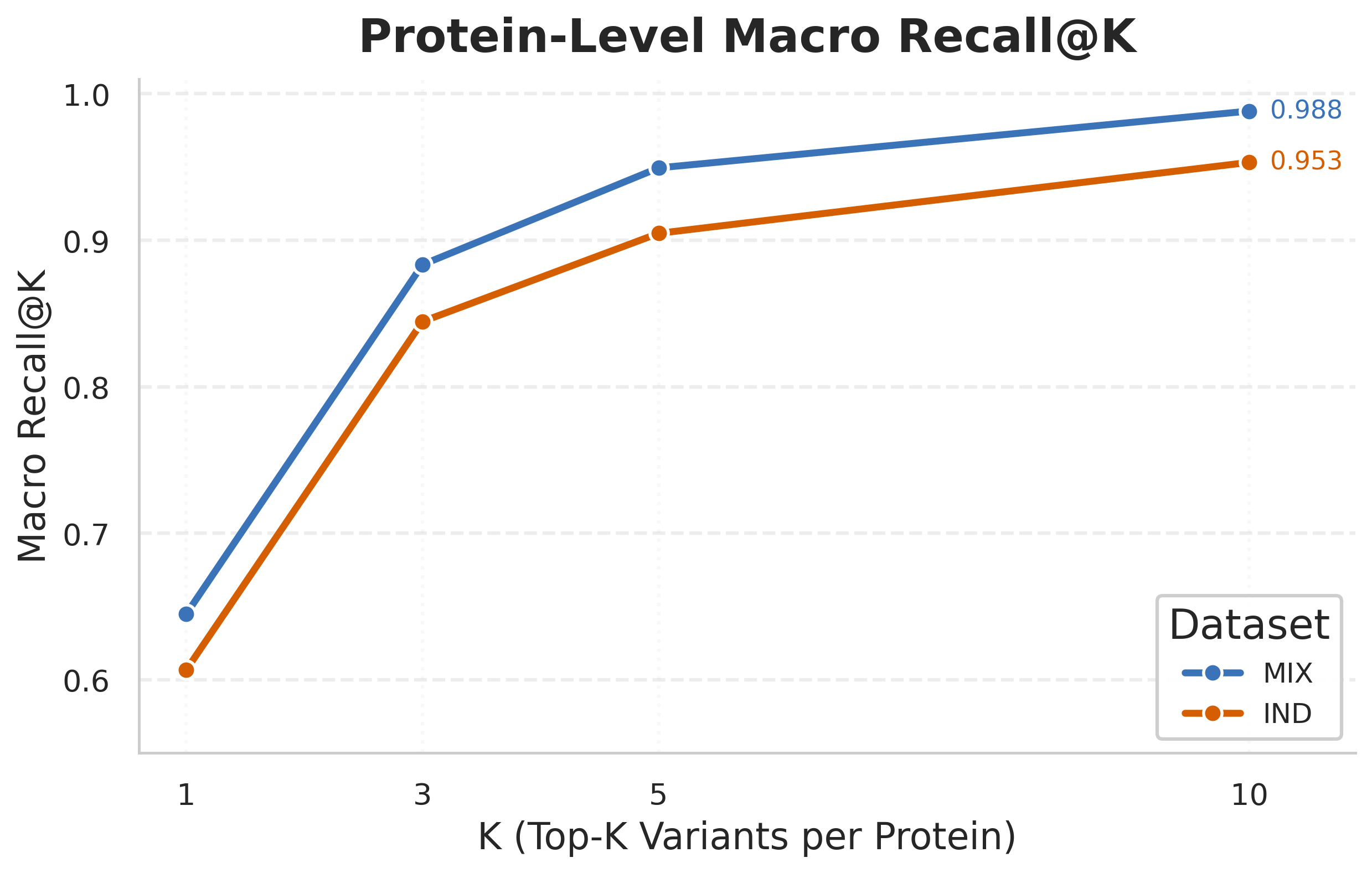
**

**Supplementary Figure 10.** Within-protein variant prioritization performance measured by protein-level macro Recall@K.


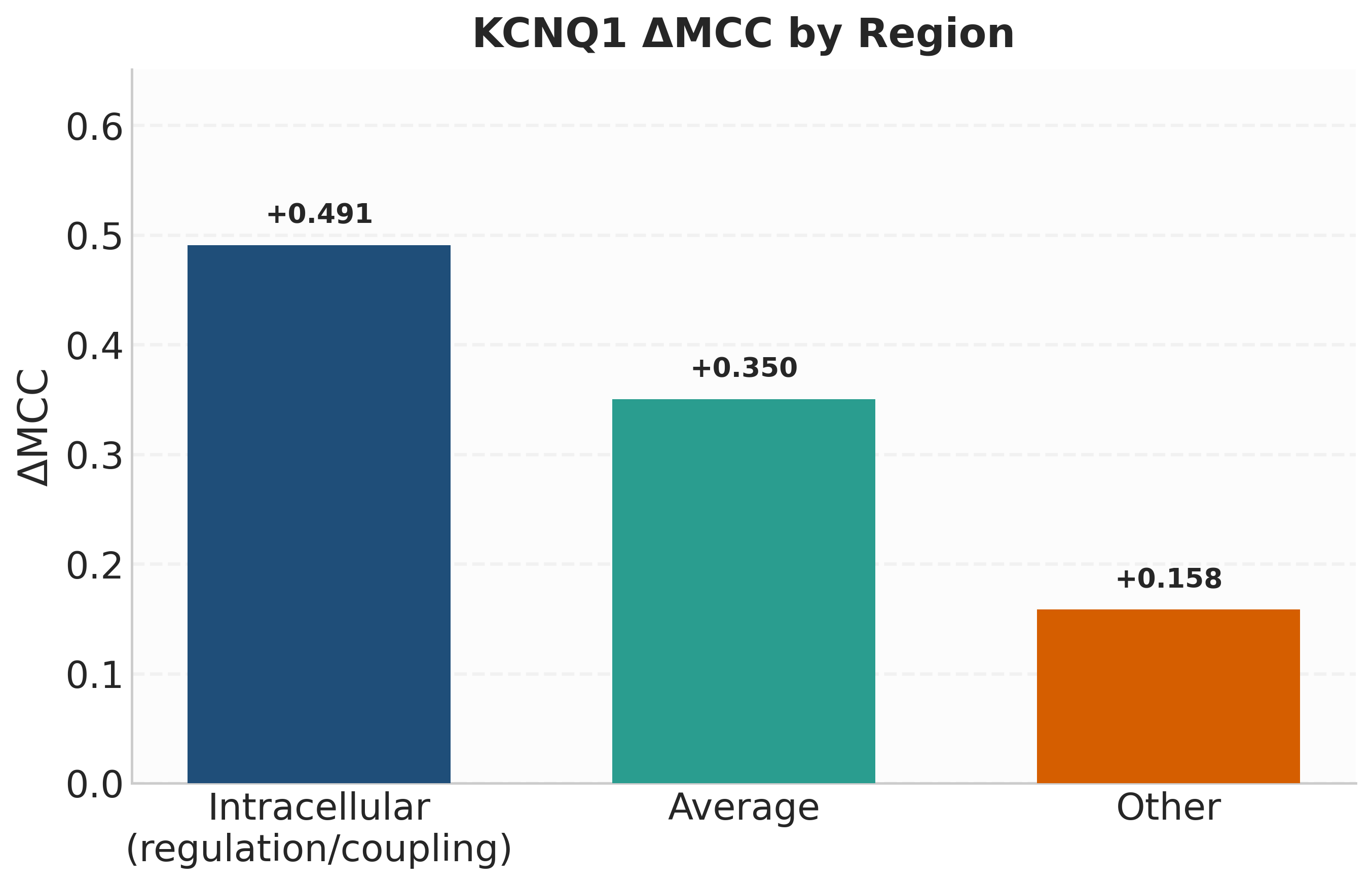


**Supplementary Figure 11.** Region-specific performance gain of Memo-Patho over AlphaMissense on KCNQ1.


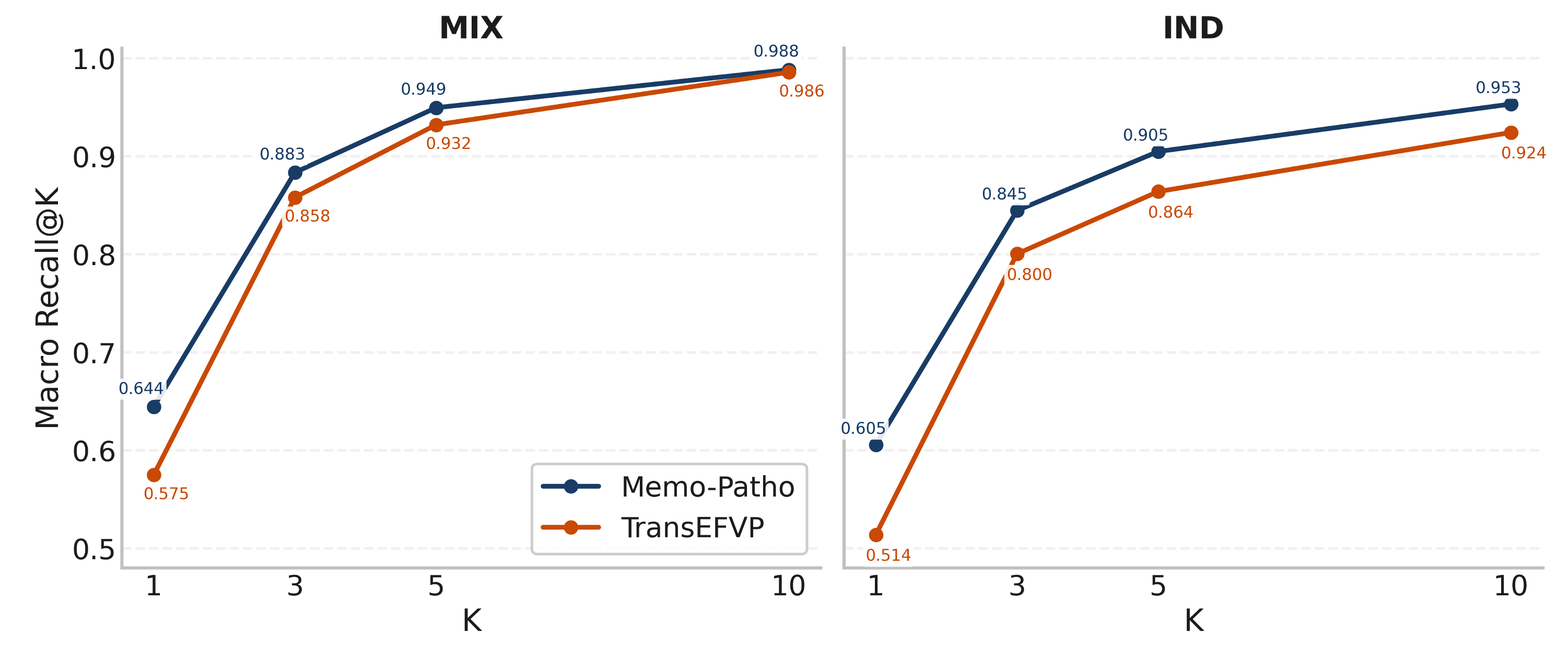


**Supplementary Figure 12.** Protein-level Recall@K of Memo-Patho and TransEFVP.


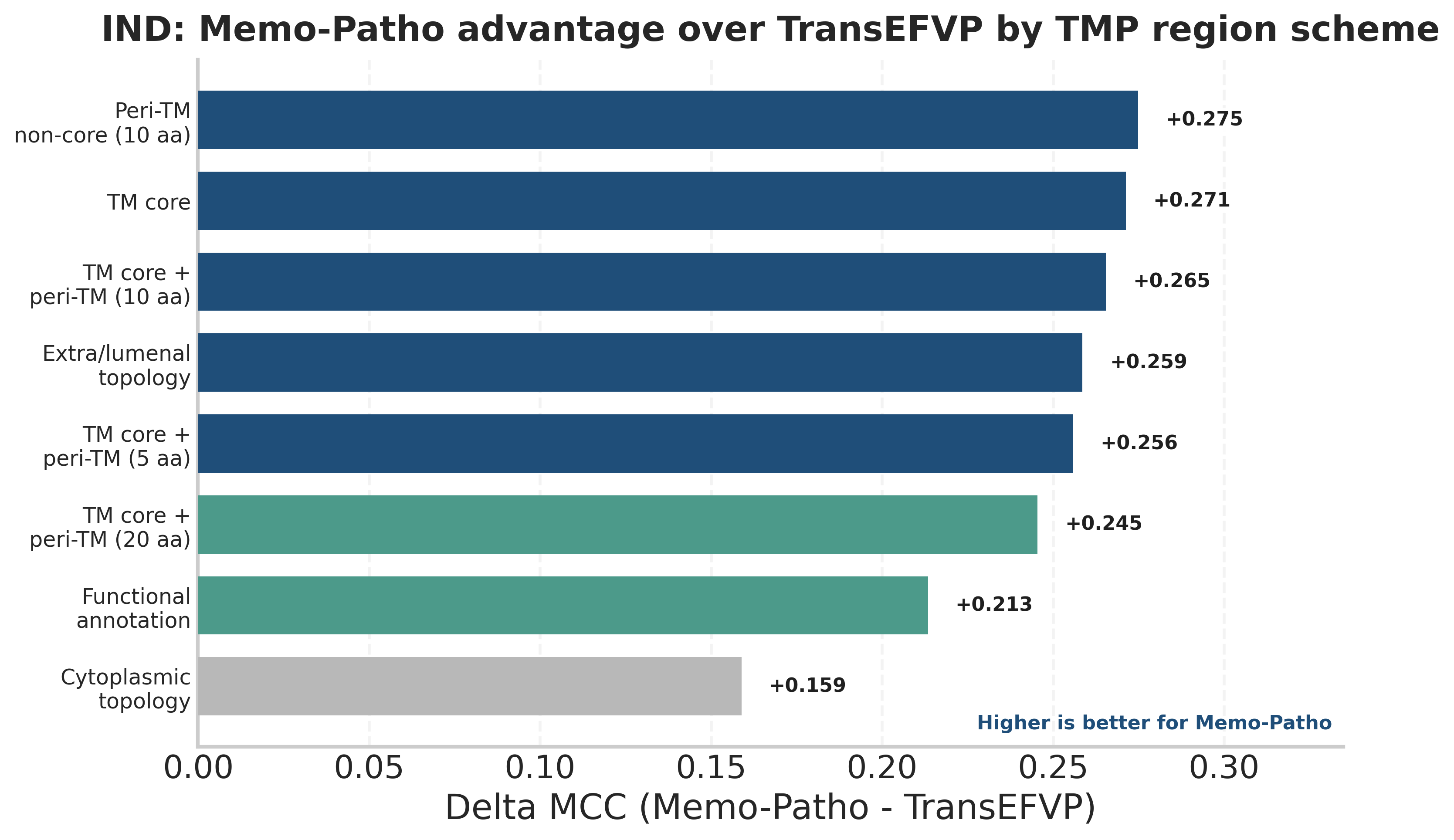


**Supplementary Figure 13.** Region-specific gain of Memo-Patho over TransEFVP.


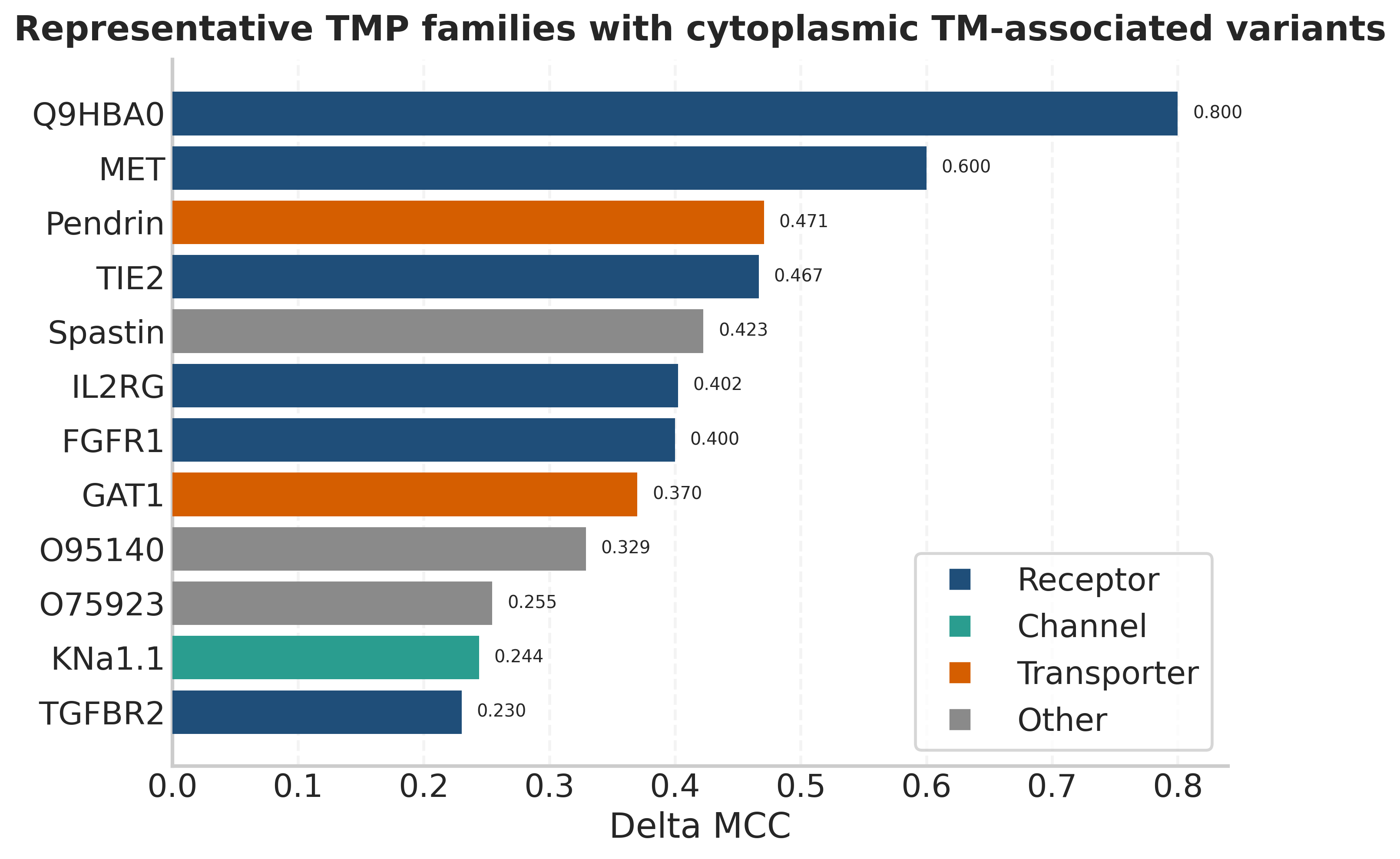


**Supplementary Figure 14.** Representative TMP families with Memo-Patho gain over TransEFVP.


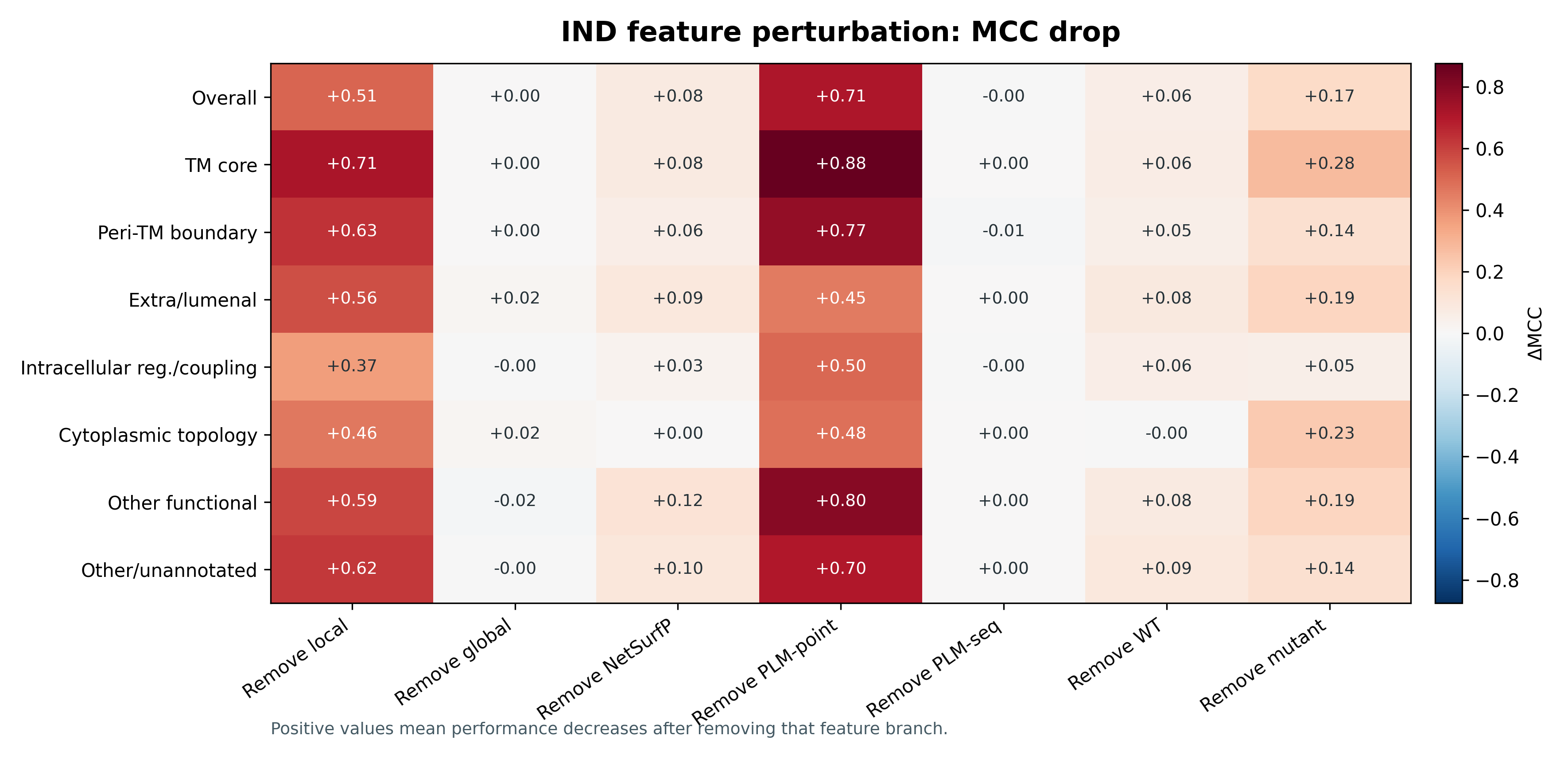


**Supplementary Figure 15.** Feature perturbation analysis of Memo-Patho on the Ind dataset.


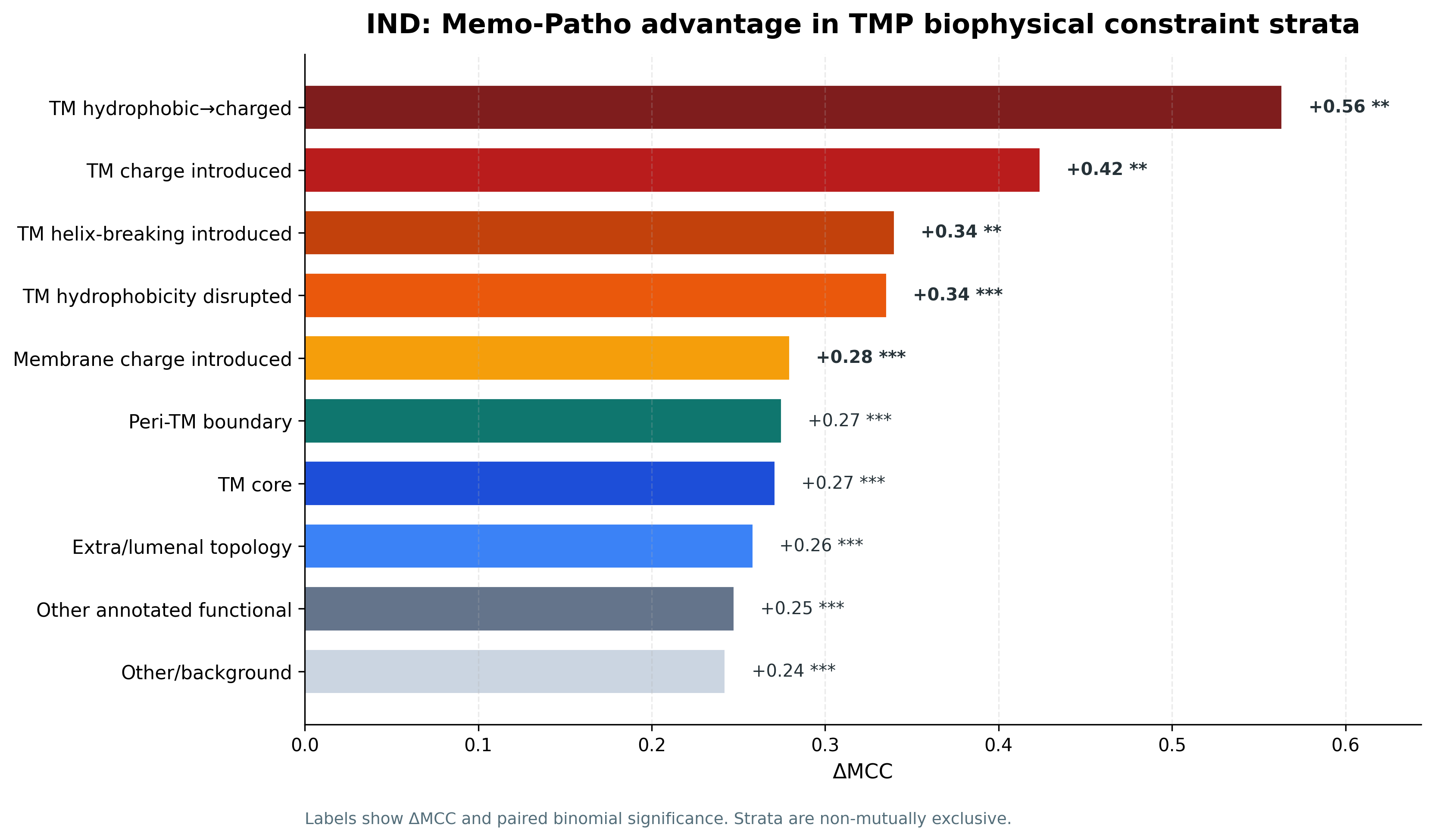


**Supplementary Figure 16.** Memo-Patho advantage across TMP biophysical constraint strata.


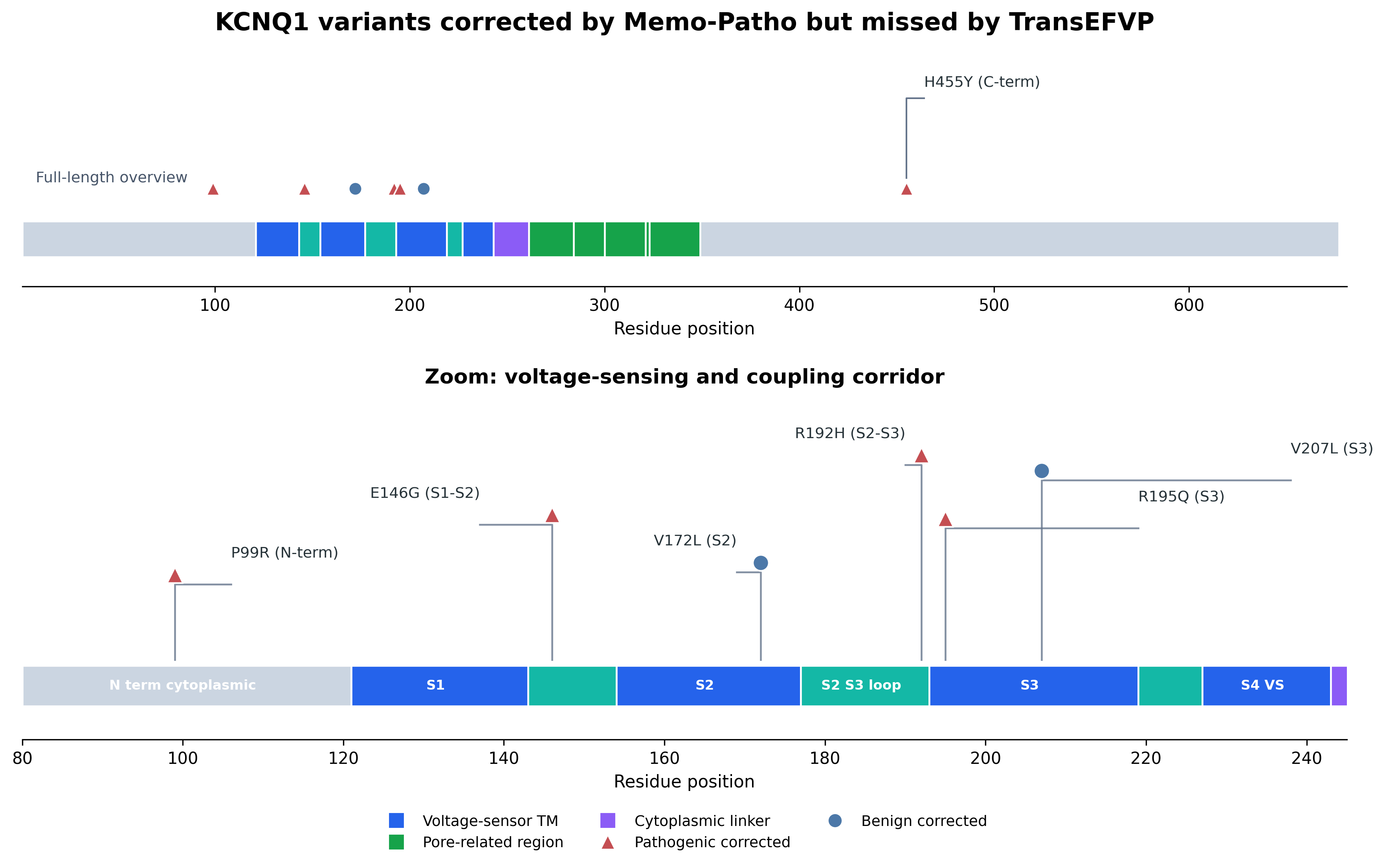


**Supplementary Figure 17.** KCNQ1 variants corrected by Memo-Patho but missed by TransEFVP.


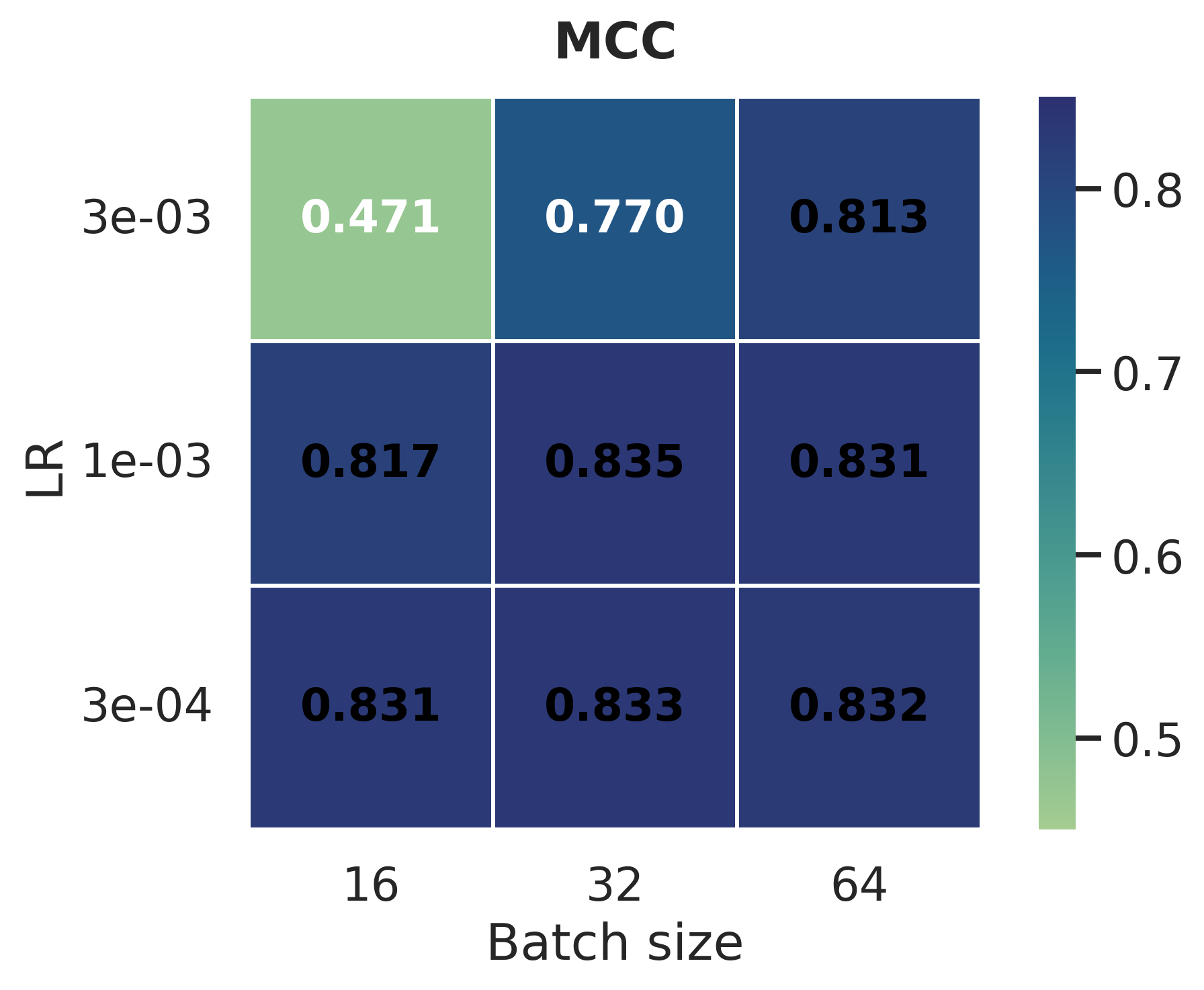


**Supplementary Figure 18.** Hyperparameter sensitivity analysis of Memo-Patho across different learning rates and batch sizes, evaluated by MCC


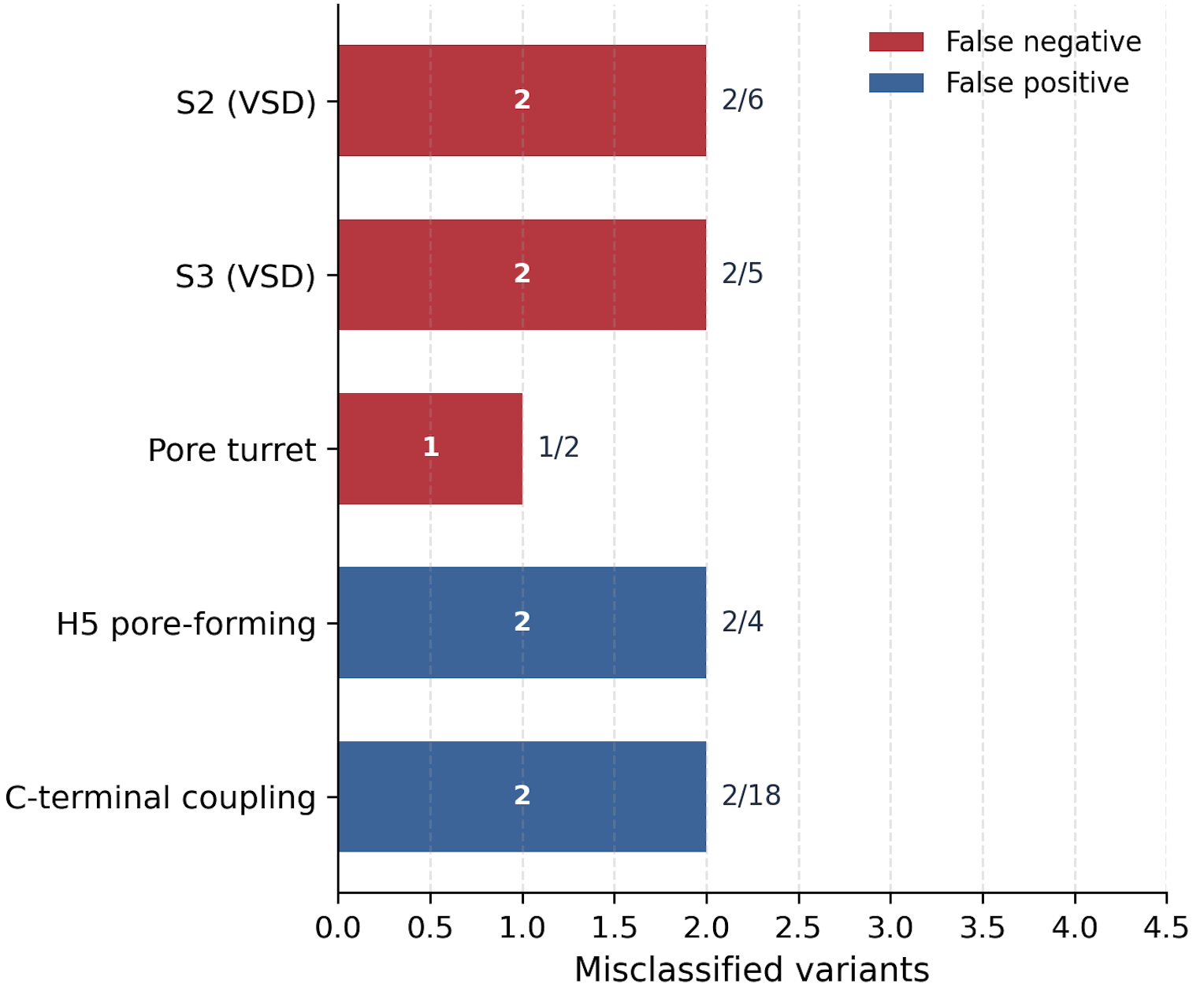


**Supplementary Figure 19.** Distribution of residual misclassified variants of Memo-Patho across structural regions in the independent KCNQ1 dataset.


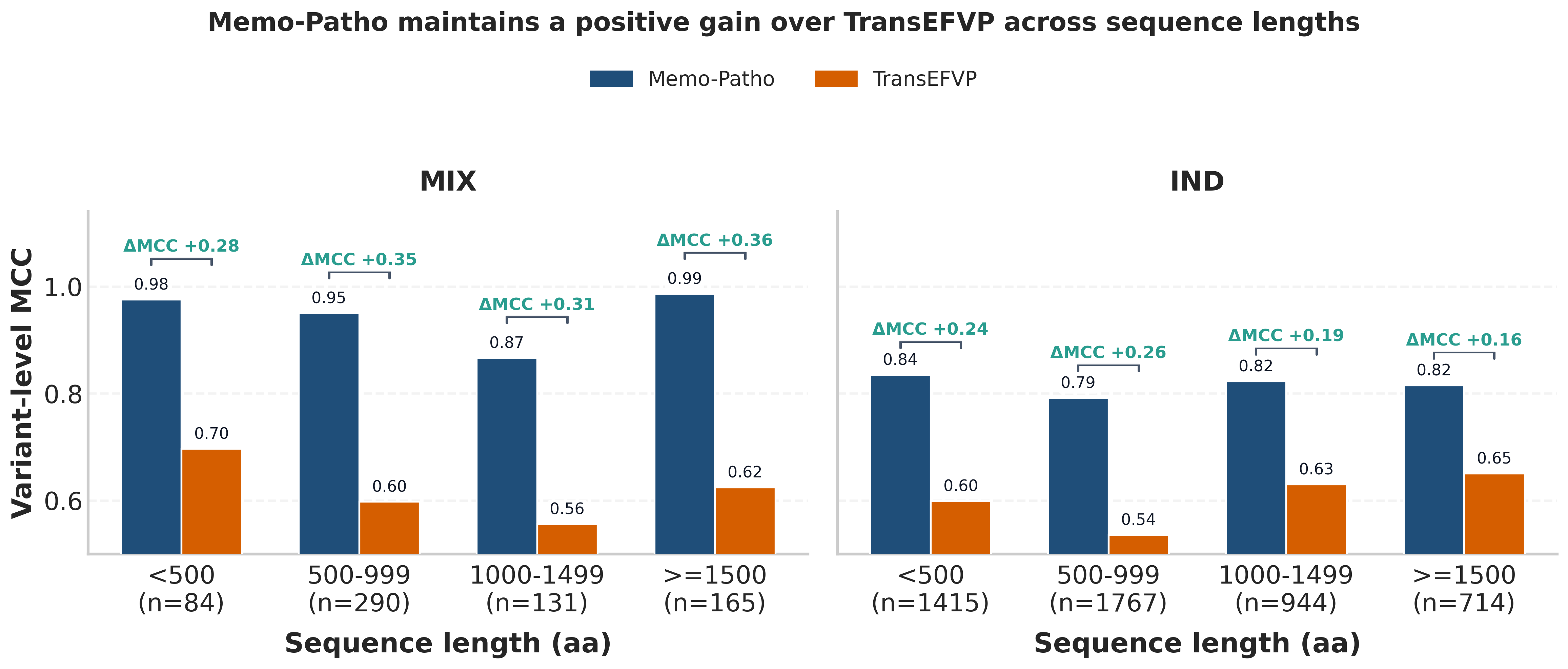


**Supplementary Figure 20.** Length-stratified performance comparison of Memo-Patho and TransEFVP.

Supplementary Note

PLMs embedding generation

1. ESM2: We employed the ESM2 protein language model in this study. The model setup and deployment strictly followed the procedures outlined in the official Facebook Research ESM GitHub repository, accessible at https://github.com/facebookresearch/esm. Specifically, the pre-trained weights corresponding to the esm2_t36_3B_UR50D version were used. These weights were obtained directly from the official distribution point: https://dl.fbaipublicfiles.com/fair-esm/models/esm2_t36_3B_UR50D.pt. This model architecture comprises 36 transformer layers and contains approximately 3 billion trainable parameters.
2. ProtT5: The ProtT5 model implementation was based on the guidelines and code provided in the ProtTrans GitHub repository (https://github.com/agemagician/ProtTrans). We utilized the ProtT5-XL-UniRef50 variant, leveraging its pre-trained weights. These weights are publicly available via the Hugging Face model repository, located at <https://huggingface.co/Rostlab/prot_t5_xl_uniref50/tree/main>.
3. Justification for Model Selection: The selection of the esm2_t36_3B_UR50D and ProtT5-XL-UniRef50 models was guided by the objective to maximize predictive performance while operating within the limitations of our computational infrastructure. These models represented the most powerful options compatible with our hardware constraints at the time of the study.
4. Transformer fusion module settings. The local-global feature fusion module used a Transformer block with one layer and eight attention heads. Positional encoding was applied based on the ordered fused feature tokens, rather than a separate TMP topology-specific positional scheme. This design was adopted because the fusion module aims to model interactions between already extracted local and global representations, while TMP-related structural and topological information is primarily provided upstream through the contextual sequence features and predicted structural descriptors.

Calculation methods for evaluation metrics

$$Accuracy= \frac{TP+TN}{TP+FN+FP+TN}$$

$$Recall= \frac{TP}{TP+FN}$$

$$Precision= \frac{TP}{TP+FP}$$

$$F1-score=2\times\frac{Recall \times Precision}{Recall+Precision}$$

$$MCC= \frac{TP\times TN-FP\times FN}{\sqrt{(TP+FP)(TP+FN)(TN+FP)(TN+FN)}}$$

Evaluation workflow for comparable tools

We evaluated its performance against several established pathogenicity prediction tools, following recommended or feasible usage protocols for each. (i) AlphaMissense: Predictions were obtained programmatically by querying the official AlphaMissense web API for specific residue positions (e.g., https://alphamissense.hegelab.org/hotspot?uid=[UniProtID]&resi=[Position]). (ii) TransEFVP: We reproduced this method locally following the released implementation and its described two-stage framework. For each variant, we extracted a fixed 201-residue sequence window centered on the mutation site (±100 residues). We then generated ten protein language model features for each sample: six ESM-based residue-level embeddings, including wild-type and mutant residue embeddings from ESM-2, ESM-1v, and ESM-1b, and four ProtT5-based embeddings, including global mean-pooled embeddings and local mutation-site embeddings for both the wild-type and mutant sequence windows. These features were organized as two inputs with shapes (6,1280) and (4,1024), respectively. In the first stage, each input branch was linearly projected to 512 dimensions and processed by a Transformer encoder with 4 layers, 8 attention heads, feed-forward dimension 1024, and dropout 0.3. The two branches were then flattened and concatenated, followed by fully connected layers of sizes 512, 256, and 128, with an additional dropout layer of 0.1 before the final sigmoid output. The model was trained using Adam (learning rate 1e-5, beta1 0.9, beta2 0.999, epsilon 1e-8) and binary focal loss (alpha 0.25, gamma 2), with batch size 1024, validation split 0.1, maximum 1000 epochs, and early stopping on validation loss with patience 20. In the second stage, the fused representation from the first-stage network was extracted, reduced using PCA with 90% retained explained variance, and then classified using an SVM with default settings in scikit-learn 1.0.2. As the released implementation did not provide an explicit hyperparameter search protocol, we used the fixed architecture and training settings specified in the public code. All code and data used for this reproduction were obtained from the public GitHub repository: https://github.com/yzh9607/TransEFVP/tree/master. (iii) E-SNPs&GO: Predictions were generated via its web server (https://esnpsandgo.biocomp.unibo.it/), which accepts full sequences without context length restrictions and supports batch input of up to 1000 mutations, including multiple mutations per protein in a single submission. (iv) PredMutHTP: Similar to E-SNPs&GO, this tool was evaluated using its web server (https://www.iitm.ac.in/bioinfo/PredMutHTP/pred.php), accepting full sequences and batch inputs of up to 1000 mutations. (v) PROVEAN: Predictions were obtained using the human species setting on its batch submission web server (http://provean.jcvi.org/protein_batch_submit.php?species=human), which processes full sequences. (vi) MutPred2: Evaluated via its web server (http://mutpred.mutdb.org/#qform), this tool accepts full sequences but limits batch submissions to a maximum of 100 mutations and a total sequence length not exceeding 35,000 residues per job. (vii) PON-P3: Predictions were obtained using the official PON-P3 web server. PON-P3 requires standardized reference-based inputs rather than flexible sequence-based input, and returns three-class predictions instead of a direct binary output. It was therefore evaluated under its supported input and output scheme. (viii) PATHOS: PATHOS was evaluated locally using the official Python implementation released by the authors. Unlike sequence-based predictors, PATHOS requires UniProt identifiers as input, which restricted evaluation to variants compatible with its input format.

For the independent KCNQ1 external validation, the revised comparison was expanded to include PON-P3 and PATHOS in addition to the representative baselines initially reported, in order to improve consistency with the broader benchmarking analysis in the main manuscript.

Memo-Patho training methods and settings

In the training phase of Memo-Patho, we defined a sequence receptive field of 501 residues centered on the mutation site. This means that for feature extraction and model input, we considered the mutated residue along with 250 residues upstream and 250 residues downstream. Utilizing such a fixed-length context window is a common practice when applying deep learning to protein sequences [1, 2]. This ensures consistent input dimensionality for the model and balances the capture of relevant local sequence information against the computational complexity and memory demands that can arise from processing entire, potentially very long, protein sequences. In the context of transmembrane proteins, this design also helps preserve broader sequence context potentially relevant to membrane organization and longer-range inter-residue dependencies, while remaining computationally tractable. It should be noted that this receptive field is used for sequence representation learning, whereas the mutation-centered structural descriptors are constructed from a compact local neighborhood around the mutated residue, allowing Memo-Patho to jointly model both broader contextual information and immediate local perturbation. To ensure robust evaluation and mitigate potential dataset biases, we employed a 10-fold cross-validation strategy during training. In this approach, the relevant training/validation dataset was randomly partitioned into 10 equally sized folds. The model was then trained 10 independent times. In each iteration, one distinct fold served as the validation set, while the remaining 9 folds were used for training. All hyperparameters were selected exclusively within the training folds and fixed before evaluation on the independent test sets, to avoid information leakage.

For model optimization, we utilized the Cross-Entropy Loss function [3] with the Adam optimizer [4]. To further assess the robustness of model optimization, we performed a targeted sensitivity analysis over these key hyperparameters. Specifically, we evaluated combinations of learning rates and batch sizes and summarized the corresponding MCC values in Supplementary Figure 18. The results showed that Memo-Patho maintained consistently strong performance across a reasonably broad range of settings. Based on these results, the final hyperparameters were set to a batch size of 32 and an initial learning rate of $1\times{10}^{-3}$. The Memo-Patho model was implemented using the PyTorch framework [5], and all training and testing experiments were conducted on a workstation equipped with three Nvidia RTX 3090 GPUs and 256GB of system RAM.

Runtime benchmarking and implementation details

To clarify the computational efficiency claim of Memo-Patho, we performed a dedicated runtime benchmark under controlled hardware settings. The benchmark was designed to separately evaluate the two main stages of deployment, namely feature generation and downstream prediction.

For Memo-Patho, the reported runtime was divided into two parts. Feature generation included the generation of PLM embeddings, and the preparation of sequence-derived structural descriptors required by the model. Prediction corresponded to running the trained Memo-Patho model on the prepared features to obtain pathogenicity predictions. Unless otherwise stated, the reported runtime reflects the directly comparable local computation stage.

To provide a realistic yet computationally manageable benchmark, we derived subsets from the Mix and Ind datasets by randomly sampling 10% of the available mutation space. The resulting benchmark subsets contained 177 proteins and 542 mutations for the Mix dataset, and 99 proteins and 331 mutations for the Ind dataset.

For the feature generation stage, we used HHblits 3.3.0 with the UniRef30_2023_02 database as a representative alignment-based preprocessing baseline, and additionally benchmarked AlphaMissense feature preparation using its official implementation. For the prediction stage, we compared Memo-Patho with the available AlphaMissense prediction code path. Because PON-P3, PROVEAN, and Pred-MutHTP are only accessible through public web servers, they were not included in the controlled runtime benchmark under identical local hardware settings.

The runtime benchmark was performed on the SJTU supercomputing cluster, with each process allocated 16 CPU cores and 1 NVIDIA A100 GPU. Memo-Patho was implemented in PyTorch 2.3.1+cu121, using a batch size of 32, 16 DataLoader workers, and FP32 precision during inference. The main training and testing experiments were originally conducted on a workstation equipped with three NVIDIA RTX 3090 GPUs and 256 GB RAM.

As summarized in Supplementary Table 5, Memo-Patho completed feature generation in 12.5 min and 11.2 min on the Mix and Ind subsets, respectively, compared with 324.5 min and 176.0 min for HHblits and more than 1440 min for AlphaMissense feature preparation. In the downstream prediction stage, Memo-Patho required less than 1 min on both subsets, whereas AlphaMissense required 767.8 min and 500.1 min, respectively. These results support the practical applicability of Memo-Patho for high-throughput pathogenicity prediction and large-scale mutational screening.

To further evaluate how Memo-Patho scales with protein length, we benchmarked representative sequences with lengths of approximately 106, 200, 299, 400, and 500 residues. The corresponding average processing times were 0.25, 0.28, 0.32, 0.38, and 0.42 s per sequence, respectively. These results indicate that runtime increases gradually with sequence length but remains efficient across the tested range. Because Memo-Patho avoids MSA generation and uses a fixed mutation-centered receptive field with a maximum effective input length of 501 residues, its runtime growth is naturally constrained, which supports its suitability for long TMPs and proteome-wide screening applications.

***Supplementary Table 1.*** *Performance evaluation between baseline models on Mix dataset*

| **Model** | **Accuracy** | **Precision** | **Recall** | **F1** | **MCC** |
| --- | --- | --- | --- | --- | --- |
| Memo-Patho | 0.9317 | 0.9318 | 0.9146 | 0.9231 | 0.8618 |
| PON-P3 | 0.8128 | 0.7678 | 0.8144 | 0.7904 | 0.6225 |
| ESNPs&GO  PROVEAN  TransEFVP  AlphaMissense  MutPred2  PredMutHTP  PATHOS  PON-P3^#^ | 0.8769  0.8001  0.8327  0.8432  0.8068  0.7695  0.8858  0.6199 | 0.9373  0.7370  0.8377  0.9207  0.7656  0.6757  0.9060  0.5524 | 0.8464  0.8255  0.7778  0.7567  0.8194  0.8868  0.8193  0.6486 | 0.8606  0.7787  0.8067  0.8057  0.7916  0.767  0.8605  0.5967 | 0.7507  0.6007  0.6611  0.6792  0.6132  0.5669  0.7670  0.2444 |
| AlphaMissense^#^ | 0.8046 | 0.7973 | 0.7308 | 0.7626 | 0.5987 |

‘^#^’ indicates we treat ‘ambiguous’ sample as wrong.

***Supplementary Table 2.*** *Performance evaluation between baseline models on Ind dataset*

| **Model** | **Accuracy** | **Precision** | **Recall** | **F1** | **MCC** |
| --- | --- | --- | --- | --- | --- |
| Memo-Patho | 0.9149 | 0.9118 | 0.8679 | 0.8893 | 0.8209 |
| PON-P3 | 0.8277 | 0.7374 | 0.8552 | 0.792 | 0.6515 |
| ESNPs&GO | 0.8932 | 0.8655 | 0.8459 | 0.8556 | 0.771 |
| PROVEAN | 0.8017 | 0.698 | 0.8167 | 0.7527 | 0.5939 |
| TransEFVP | 0.8117 | 0.8272 | 0.6601 | 0.7343 | 0.6004 |
| AlphaMissense | 0.8666 | 0.8692 | 0.7700 | 0.8166 | 0.7156 |
| PredMutHTP  PATHOS | 0.7716  0.8793 | 0.6517  0.8654 | 0.8729  0.8097 | 0.7462  0.8367 | 0.5658  0.7421 |
| PON-P3^#^ | 0.5999 | 0.4849 | 0.6955 | 0.5714 | 0.2306 |
| AlphaMissense^#^ | 0.8205 | 0.7840 | 0.7378 | 0.7602 | 0.6177 |

‘^#^’ indicates we treat ‘ambiguous’ sample as wrong.

***Supplementary Table 3.*** *Performance evaluation between baseline models on novel dataset*

| **Model** | **Accuracy** | **Precision** | **Recall** | **F1** | **MCC** |
| --- | --- | --- | --- | --- | --- |
| Memo-Patho | 0.839 | 0.855 | 0.870 | 0.862 | 0.668 |
| ESNPs&GO | 0.729 | 0.681 | 0.970 | 0.800 | 0.484 |
| TransEFVP | 0.797 | 0.839 | 0.788 | 0.813 | 0.592 |
| AlphaMissense | 0.678 | 0.750 | 0.636 | 0.689 | 0.365 |
| PredMutHTP  PATHOS  PON-P3  PON-P3^#^ | 0.661  0.796  0.624  0.527 | 0.640  0.872  0.612  0.558 | 0.941  0.759  0.963  0.889 | 0.762  0.812  0.748  0.686 | 0.304  0.598  0.206  -0.160 |
| AlphaMissense^#^ | 0.661 | 0.724 | 0.636 | 0.677 | 0.326 |

‘^#^’ indicates we treat ‘ambiguous’ sample as wrong.

***Supplementary Table 4.*** *Computational efficiency of different PLM configurations*

| **Split** | **Model** | **Trainable Parameters (M)** | **Embedding Time (s/variant)** | **Classifier Inference Time (ms/sample)** |
| --- | --- | --- | --- | --- |
| Mix | Full | 13.35 | 0.314 | 0.722 ± 0.009 |
| Mix | ESM2-only | 11.26 | 0.252 | 1.022 ± 0.052 |
| Mix | ProtT5-only | 8.11 | 0.062 | 1.023 ± 0.066 |
| Ind | Full | 13.35 | 0.309 | 0.744 ± 0.026 |
| Ind | ESM2-only | 11.26 | 0.249 | 1.153 ± 0.044 |
| Ind | ProtT5-only | 8.11 | 0.060 | 1.117 ± 0.055 |

***Supplementary Table 5.*** *Runtime benchmark of Memo-Patho and representative baseline pipelines under controlled hardware settings*

| **Dataset** | **Task** | **Method** | **Time (min)** |
| --- | --- | --- | --- |
| Mix  (10%) | Feature generation | HHblits | 324.5 |
|  |  | AlphaMissense | >1440 |
|  |  | Memo-Patho | 12.5 |
|  | Prediction | AlphaMissense | 767.8 |
|  |  | Memo-Patho | <1 |
| Ind  (10%) | Feature generation | HHblits | 176.0 |
|  |  | AlphaMissense | >1440 |
|  |  | Memo-Patho | 11.2 |
|  | Prediction | AlphaMissense | 500.1 |
|  |  | Memo-Patho | <1 |

Reference

[1] Z. Liu, Y. Gong, Y. Bao, Y. Guo, H. Wang, and G. N. Lin, "TMPSS: a deep learning-based predictor for secondary structure and topology structure prediction of alpha-helical transmembrane proteins," *Frontiers in bioengineering and biotechnology,* vol. 8, p. 629937, 2021.

[2] F. Palhamkhani, M. Alipour, A. Dehnad, K. Abbasi, P. Razzaghi, and J. B. Ghasemi, "DeepCompoundNet: enhancing compound–protein interaction prediction with multimodal convolutional neural networks," *Journal of Biomolecular Structure and Dynamics,* vol. 43, no. 3, pp. 1414-1423, 2025.

[3] A. Mao, M. Mohri, and Y. Zhong, "Cross-entropy loss functions: Theoretical analysis and applications," in *International conference on Machine learning*, 2023: PMLR, pp. 23803-23828.

[4] Z. Zhang, "Improved adam optimizer for deep neural networks," in *2018 IEEE/ACM 26th international symposium on quality of service (IWQoS)*, 2018: Ieee, pp. 1-2.

[5] S. Imambi, K. B. Prakash, and G. Kanagachidambaresan, "PyTorch," *Programming with TensorFlow: solution for edge computing applications,* pp. 87-104, 2021.
